# The old world *salsola* as a source of valuable secondary metabolites endowed with diverse pharmacological activities: a review

**DOI:** 10.1080/14756366.2022.2102005

**Published:** 2022-07-25

**Authors:** Mai H. ElNaggar, Wagdy M. Eldehna, Mohammed A. S. Abourehab, Fatma M. Abdel Bar

**Affiliations:** a Department of Pharmacognosy, Faculty of Pharmacy, Kafrelsheikh University, Kafrelsheikh, Egypt; b Department of Pharmaceutical Chemistry, Faculty of Pharmacy, Kafrelsheikh University, Kafrelsheikh, Egypt; c Department of Pharmaceutics, Faculty of Pharmacy, Umm Al-Qura University, Makkah, Saudi Arabia; d Department of Pharmaceutics and Industrial Pharmacy, Faculty of Pharmacy, Minia University, Minia, Egypt; e Department of Pharmacognosy, College of Pharmacy, Prince Sattam Bin Abdulaziz University, Al-Kharj, Saudi Arabia; f Department of Pharmacognosy, Faculty of Pharmacy, Mansoura University, Mansoura, Egypt

**Keywords:** Genus *Salsola*, phytochemicals, traditional uses, biological activity, enzyme inhibition

## Abstract

*Salsola* is an important genus in the plant kingdom with diverse traditional, industrial, and environmental applications. *Salsola* species are widely distributed in temperate regions and represent about 45% of desert plants. They are a rich source of diverse phytochemical classes, such as alkaloids, cardenolides, triterpenoids, coumarins, flavonoids, isoflavonoids, and phenolic acids. *Salsola* spp. were traditionally used as antihypertensive, anti-inflammatory, and immunostimulants. They attracted great interest from researchers as several pharmacological activities were reported, including analgesic, antipyretic, antioxidant, cytotoxic, hepatoprotective, contraceptive, antidiabetic, neuroprotective, and antimicrobial activities. Genus *Salsola* is one of the most notorious plant genera from the taxonomical point of view. Our study represents a comprehensive review of the previous phytochemical and biological research on the old world *Salsola* secies. It is designed to be a guide for future research on different plant species that still belong to this genus or have been transferred to other genera.

## Graphical Abstract

**Figure:**
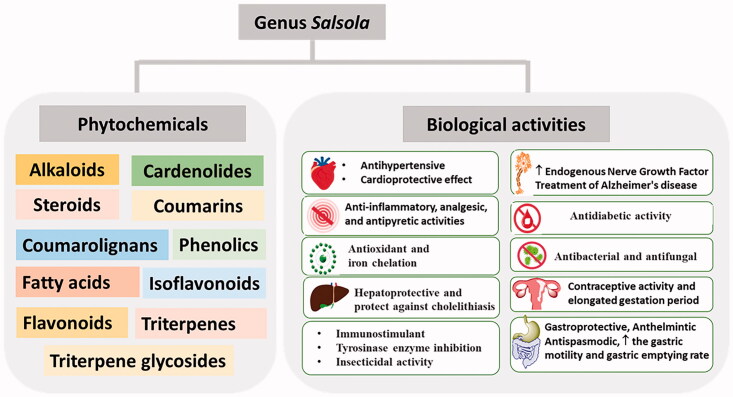

## 1. Introduction

Plants are considered as a latent treasure and a vital source for the discovery of medicines. They include a plethora of secondary metabolites that act as modulators for the enzymes involved in human diseases^1^^,^^2^. Plant extracts and their derived natural products or analogues are extensively reported to exert promising effects on human devastating diseases including different types of cancer^3–6^. They are also reported to protect humans against different types of microbes^7^ and recently evolved infectious diseases as COVID-19^8^^,^^9^.

The genus *Salsola* (commonly known as saltwort) belongs to the family Amaranthaceae, previously Chenopodiaceae. The genus name is from the Latin words “salsus” or “sallere” meaning salty because they are halophytes capable of living in saline environments or due to their content of alkaline salts, such as potassium and sodium carbonates^10–12^. The old genus *Salsola* comprised about 150 sp. growing in extreme climatic conditions as arid, semi-arid, and temperate regions worldwide^11^^,^^13^. They represented about 45% of the desert plants^11^ and some of them are invasive species^14^. Various plants of the genus *Salsola* are edible and some of them have been used in traditional medicine^15^. Some of them are also reported to be rich in fibre content^16^. They have important value as animal feed and they are beneficial in the reclamation and phytoremediation of soil contaminated with heavy metals^11^^,^^14^. Plants belonging to this genus also represent a rich source for endophytic microbes that could be used for potential biological applications^17^^,^^18^. Furthermore, different plants of the genus *Salsola* were reported to have industrial value as the use of *S. soda* and *S. kali* as a source of sodium carbonate, in linin, and cotton bleaching, and in glass and soap making^14^^,^^19^^,^^20^.

Despite the importance of plants belonging to the genus *Salsola*, they do not receive great research attention. Most of the research is done on the respiratory diseases and the hypersensitivity caused by the pollen grains of some *Salsola* spp. and developing vaccines for it^21–23^. Very limited reviews are made on the genus *Salsola* such as the one made by Altay and Ozturk^11^ that discuss its fodder value. Hanif et al.^14^ discussed the environmental, industrial, and traditional uses of *Salsola* spp. and they mentioned a small fraction of the biological studies made on them. This article addresses almost all the research articles concerning the phytochemistry and the biological activity of the plants belonging to the old genus *Salsola* until 2021.

## 2. Morphological characters

**Figure 1.**
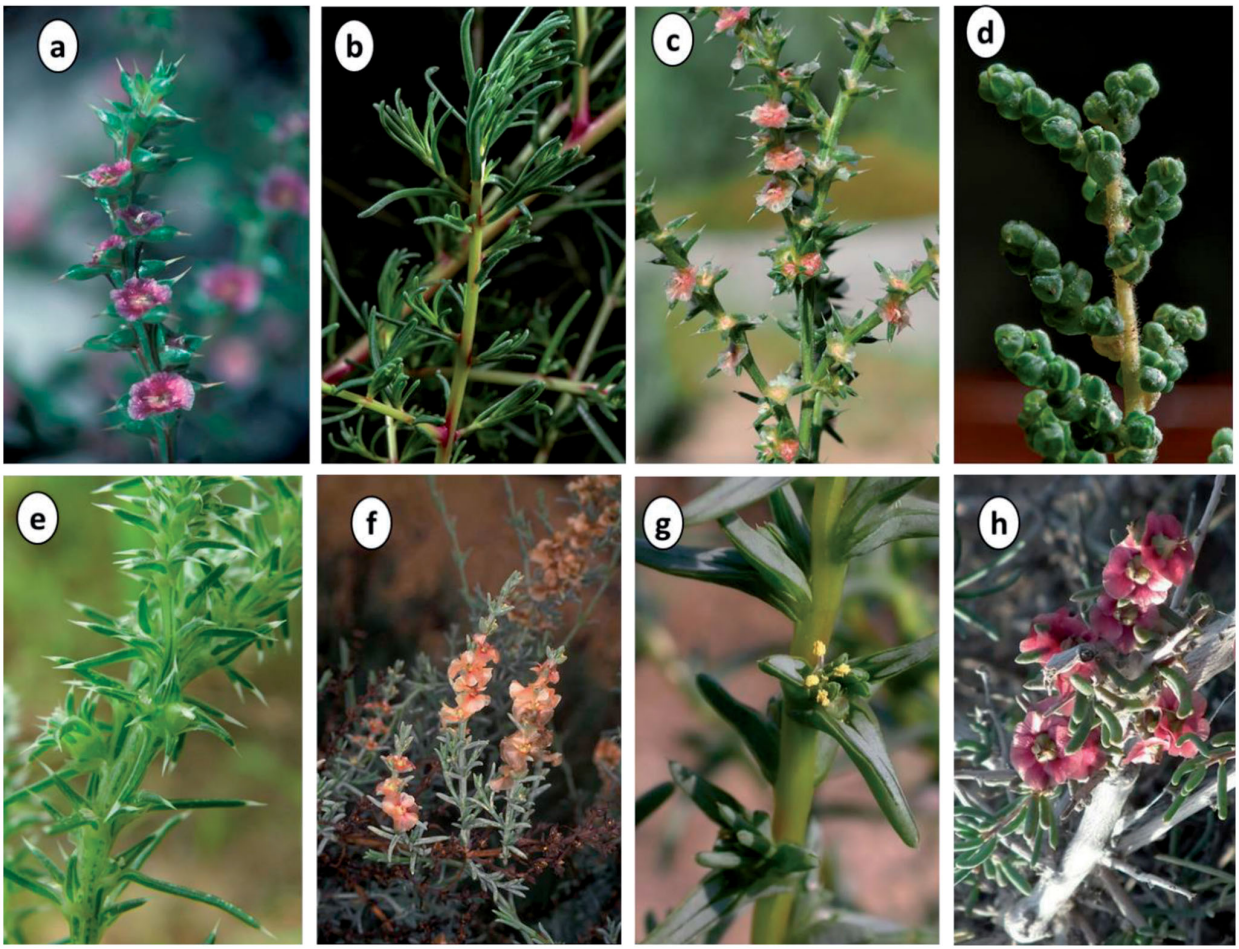
Photographs of selected Salsola spp.; **a.** *S. kali* (adapted from kali https://gobotany.nativeplanttrust.org/sp./salsola/kali/), **b.** *S. collina*, **c.** *S. tragus*, **d.** *S. imbricata* (adapted from https://www.floraofqatar.com/amaranthaceae.htm), **e.** *S. komarovii*, **f.** *S. oppositifolia* Desf. (adapted from adapted from https://powo.science.kew.org/), **g**. *S. soda* (adapted from https://eunis.eea.europa.eu/sp./168053), **h.** *S. laricifolia* (adapted from https://panama.inaturalist.org/taxa/985676-Salsola-laricifolia).

Members of the genus *Salsola* are shrubs, sub-shrubs, annual or perennial herbs. They are characterised by small, sessile, often succulent leaves that may be opposite or alternate. Most have bisexual axillary flowers that can be solitary or clustered to form loose or dense spikes (Figure 1). Each flower is subtended by two prominent bracteoles, with a frequently hard 5-segmented perianth (often winged in fruit), and a superior ovary. Seeds are horizontal, subglobose, with a spiral embryo^11^^,^^24^^,^^25^.

## 3. Taxonomic classification

Genus *Salsola* belongs to the flowering plant family Amaranthaceae descending from the order Caryophyllales^26^. *Salsola* has a long history of being considered as one of the largest genera within the family Chenopodiaceae containing 100 to 190 sp.^27^. While it is classified now as one of the Amaranthaceae genera after merging family Chenopodiaceae with the family Amaranthaceae according to the angiosperm phylogeny group (AGP-IV)^26^^,^^28–30^. Plants belonging to the genus *Salsola* have the following taxonomic classification^27^^,^^30–32^.

Kingdom: Plantae - Plants
Subkingdom: Tracheobionta - Vascular plants
Superdivision: Spermatophyta - Seed plants
Division: Magnoliophyta - Flowering plants
Class: Magnoliopsida - Dicotyledons
Subclass: Caryophyllidae
Order: Caryophyllales
Family: Amaranthaceae (previously, Chenopodiaceae)
Subfamily: Salsoloideae
Tribe: Salsoleae
Genus: Salsola

The taxonomy of *Salsola* spp. is debateable and confusing due to their diversity and distribution in the Asian and the middle east deserts that lead to difficulties in their collection and investigation^31^. The close relationship between *Salsola* spp. and the dependence on minor morphological differences in their old classification together with the recent use of molecular techniques in plant systematics led to major changes in the classification of the genus *Salsola*^27^. The classification of the genus *Salsola* has been revised by Akhani et al. (2007) and it was spitted into 10 different genera. The transfer of different sp. from the old world *Salsola* to other genera, such as *Caroxylon* genus resulted in decreasing the number of its sp. to 25^27^.

The type of the genus *Salsola* was *Salsola soda*^27^^,^^31^, which has been recently changed by the International Code of Nomenclature into *Salsola Kali* as suggested by Mosyakin et al.^33^. This resulted in changing the name of many traditionally known *Salsola* spp. into *Soda*^28^.

These taxonomical and nomenclatural changes together with the presence of different synonyms for several *Salsola* spp. would obscure the determination of the phytochemical constituents and the biological activities of the old world *Salsola* species.

Therefore, in this article, we will review the phytochemical content and the biological activities of the old world *Salsola* spp. and indicate their current taxonomic status as illustrated in Table 1.

**Table 1.** Current taxonomic status and synonyms of *Salsola* plants mentioned in this review article.

Plant	Genus	Basionym and synonyms according to POWO^34^ and IPNI^35^	Native Distribution range^34^
*S. arbuscula*	*Xylosalsola* Tzvelev	Synonyms: *S. arborescens* *S. exasperate* *S. transhyrcanica*	European Russia to Mongolia and Pakistan
*S. collina*	*Salsola*	Basionym of *Kali collinum*^31^ Synonyms: *S. chinensis* Gand. *S. erubescens* Schrad. *S. ircutiana* Gand. *S. kali* subsp. *collina* (Pall.)	South European Russia to Korea
*S.* *cyclophylla*	**Transferred to Genus *Caroxylon*** ^31^	Basionym of *Caroxylon cyclophyllum* (Baker)^31^	Syria to Sudan and South Pakistan
*S. grandis*	*Salsola*	Basionym for *Soda grandis*^28^	Turkey
*S. imbricata*	**Transferred to Genus *Caroxylon*** ^31^	Basionym of *Caroxylon imbricatum*^31^ Synonyms: ***S. baryosma*** Schult. *Caroxylon foetidum* Moq. *Nitrosalsola baryosma* (Schult.) Theodorova *S. marosteum* Moq. *S. moorcroftiana* Wall. *Chenopodium baryosmon* Schult. ***S. foetida*** Del.^36^	Sahara & Sahel to west India distributed throughout warm desert areas of northwest India, Pakistan, Iran, Afghanistan and tropical east Africa^36^
*S. inermis* Forssk	**Transferred to Genus *Caroxylon*** ^31^	Basionym of *Caroxylon inermis* (Forssk.)^31^	Egypt, Arabian Peninsula, and Iran
*S. kali*	*Salsola*	It has different varieties and synonyms such as *S. scariosa, S. spinosa, S. turgida*	Atlantic and Mediterranean coast countries
*S. komarovii*	*Salsola*	Basionym of *Kali komarovii* (Iljin)^31^	It grows in sand dunes and beaches in Japan, China, and Korea^37^
*S. laricifolia* Turcz	*Salsola*	–	Central Asia to Mongolia and North Xinjiang
*S. longifolia* Forssk.	*Salsola*	Basionym of *Soda longifolia* (Forssk.)^28^ Synonyms as *S. fruticosa* Cav. *S. longiflora* J.F.Gmel. *S. oppositifolia* Sieber ex Moq.	Sahara to Arabian Peninsula
*S. micranthera*	***Caroxylon***	Basionym of *Caroxylon micrantherum* (Botsch.) *Nitrosalsola micranthera* (Botsch.)^38^	Central Asia to Southern Xinjiang
*S. oppositifolia* Desf.	*Salsola*	Basionym of *Soda oppositifolia* (Desf.)^28^ Synonyms: *S. oppositifolia* f. *feminea* Botsch. *Seidlitzia oppositifolia* (Desf.) Iljin	Mediterranean countries
*S. richteri*	*Xylosalsola* Tzvelev	Synonyms: *Xylosalsola richteri* (Moq.) *Salsola arborescens* var. richteri Moq.	Central Asia and Pakistan
*S. rigida* Pall.	***Caroxylon***	Synonyms: *Caroxylon orientale* Salsola orientalis S.G.Gmel. *Salsola syriaca* Botsch. *Salsola heliaramiae* Mouterde	Central Sinai to North Xinjiang and West Pakistan
*S. soda* L.	*Salsola*	Its name has been modified to *Soda inermis*^28^ Synonyms: *Salsola longifolia* Lam.	Growing on saline soils throughout Armenia, Iran, Turkey, and Turkmenistan, is cultivated and highly prized as a leaf vegetable (agretti) in the Mediterranean region
*S. somalensis*	*Halothamnus* Jaub. & Spach	Basionym of *Halothamnus somalensis*	Tropical Africa
*S. tetrandra*	**Transferred to Genus *Caroxylon*** ^31^	Basionym of *Caroxylon tetrandrum* (Forssk.)	North Africa, Palestine, Saudi Arabia, Sinai
*S. tetragona*	*Caroxylon*	Synonyms: *Caroxylon tetragonum* *Salsola pachoi* Volkens & Asch. *Salsola diplantha* Botsch *Halogeton tetragonus* (Delile) Moq.	North Africa to Palestine
*S. tragus*	*Salsola*	Basionym of *S. kali* var. tragus (L.) Moq. *S. kali* subsp. tragus (L.) Čelak. *S. ruthenica* var. tragus (L.) Morariu Synonyms as ***S. ruthenica*** *S. pestifer* A.Nelson	Europe to Siberia and Korea
*S. tuberculatiformis*	*Caroxylon*	Basionym of *Caroxylon tuberculatiforme* (Botsch.)^39^ Synonyms: *S. tuberculate*^40^	Cape, South Africa
*S. villosa* Schult.	*Caroxylon*	Synonyms as *Salsola palaestinica* Botsch. *Salsola mandavillei* Botsch. *Salsola libyca* Botsch.v*Salsola delileana* Botsch. *Salsola damascena* Botsch. *Nitrosalsola palaestinica* (Botsch.) Theodorova	Egypt, India, Lebanon-Syria, Libya, Palestine, Saudi Arabia, Sinai
*S. volkensii*	*Caroxylon/Nitrosalsola*	Basionym of *Caroxylon volkensii* (Schweinf. & Asch.)^31^ Basionym of *Nitrosalsola volkensii* (Schweinf. & Asch.)^38^	Egypt, Iraq, and Arabian Peninsula

## 4. Chemistry

### 4.1. Volatile constituents

Hexahydro-farnesyl acetone and benzoic acid esters were reported as the major constituents of *S. cyclophylla* volatile oil^15^^,^^41^. However, GC analysis of the volatile fractions of different parts of *S. vermiculate* L. plant revealed that carvone and β-caryophylline were the major components in leaves (52.2% and 5.8%, respectively), while carvone and cuminaldehyde were the major components in roots (49.9% and 4.4%, respectively). Additionally, carvone, limonene, and linalool were detected as the major constituents of the stems of *S. vermiculate* L. (53%, 17.4%, and 11.3%, respectively)^42^.

#### 4.1.1. Non-volatile constituents

Previous phytochemical investigations of plants belonging to the genus *Salsola* indicates the presence of diverse groups of secondary metabolites, such as alkaloids^43–49^, cardenolides and steroids^50^^,^^51^, coumarins and coumarolignans^52^, fatty acids^50^^,^^51^^,^^53^, flavonoids and isoflavonoids^54–59^, phenolics^60^, and triterpene glycosides^61–64^.

#### 4.1.2. Alkaloids and nitrogenous compounds

**Figure 2.**
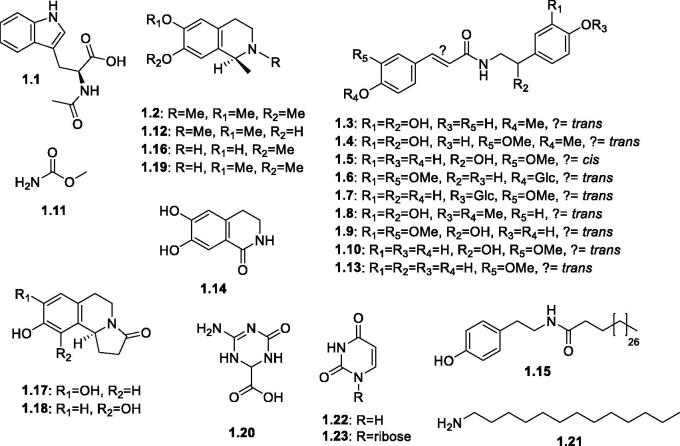
Structures of alkaloids and nitrogenous compounds (**1.1–1.23**) reported in the genus *Salsola.*

Different classes of alkaloids and other nitrogenous compounds have been reported from plants of the genus *Salsola*, Figure 2. A unique group of optically active *l*-methyl-tetrahydro-isoquinoline alkaloids have been early detected by Proskurnina and Orekhov^45^ from *Salsola richteri* Karel and the isolated alkaloids were identified as carnegine **1.2**, salsoline **1.16**, and *N*-norcarnegine (salsolidine) **1.19**. The southern Turkmenistan *salsola*, S. *richteri* Karel yielded 0.16​% of salsoline^44^. A fourth related derivative, *N*-methylisosalsoline **1.12**, was detected by GC/MS in the aerial parts of *S. oppositofolia*, *S. soda* and *S. tragus*^65^. In addition, 3,4-dihydro-6,7-dihydroxy-1(2*H*)-isoquinolinone; namely pericampylinone-A (iseluxine) **1.14**, was also isolated from *S. collina* Pall.^66^. The presence of optically active (-) pyrrolo[2,1-a]isoquinoline type alkaloids has been reported from *S. collina* Pall.^43^^,^^45^^,^^47–49^. Particularly, Zhao and Ding^47^ isolated and identified the first alkaloid of this group namely, salsoline A (trolline) **1.17**; (*S*)-8,9-dihydroxy-1,2,5,6-tetrahydropyrrolo[2,1-*a*]isoquinolin-3(10*bH*)-one followed by Xiang et al.^43^ who were able to isolate and identify another related positional isomer namely; salsoline B **1.18** from the same plant. Another group of nitrogenous derivatives, moupinamides has been reported from different *Salsola* spp. in both free and combined (glucoside) forms. They possess a skeleton of *N*-*trans*-feruloyltyramine or *N*-*trans*-feruloyldopamine structures. The structures of *N-trans-*feruloyl-3-*O*-methyldopamine **1.9** and *N*-*trans*-feruloyl-3′″-methoxydopamine 4′-*O*-β-D-glucopyranoside **1.6**, were reported in *S. collina*^43^ whereas, *N-trans-*feruloyltyramine **1.13** and 7′-hydroxy *N*-*trans*- feruloyltyramine **1.10**, were found in *S. collina* and *S. tetrandra*^43^^,^^53^^,^^66^. Also, *trans*-*N*-feruloyl tyramine-4′″-*O*-β-D-glucopyranoside **1.7**, was reported from *S. inermis* Forssk^51^. The only reported moupinamide derivative with a "*cis*" double bond configuration of the cinnamoyl moiety was *cis*-*N*-feruloyltyramine **1.5** which was isolated from the aerial parts of *S. baryosoma*^67^. It is worth noting that several tentatively (incompletely) defined structures were reported by UPLC/qTOF-MS analysis of the aerial parts and roots of *S. vermiculata* and *S. tetrandra*^68^. They included *N*-caffeoyl tyramine, *N*-(3′,4′-dimethoxy-cinnamoyl)-norepinephrine, *N*-(4′-methoxy-cinnamoyl)-norepinephrine, *N*-feruloyl-3′″-methoxytyramine However, further spectral analysis, such as 1 D and 2 D NMR are required to confirm their structures.

Another miscellaneous group of nitrogenous compounds was reported from different *Salsola* spp., including simple nitrogenous compounds, such as methyl carbamate **1.11** from *S. tetrandra*, *S. kali*, *S. longifolia* and *S. rigida*^69^. The amino acid derivative, *N*-acetyltryptophan **1.1** was isolated from *S. collina* Pall. and *S. grandis* Freitag, Vural & Adiguzel^66^^,^^70^. Pericampylinone-A **1.14**, terrestric acid **1.20**, uracil **1.22**, and uridine **1.23** were reported by Jin et al.^66^ from *S. collina* Pall. While salisomide **1.15** was reported by Saleem et al.^57^ from *S. imbricata* Forssk. The alkylamine, tridecanamine **1.21,** was also reported from the aerial parts of *S. terrandra* Forssk^71^.

#### 4.1.3. Cardenolides and steroids

**Figure 3.**
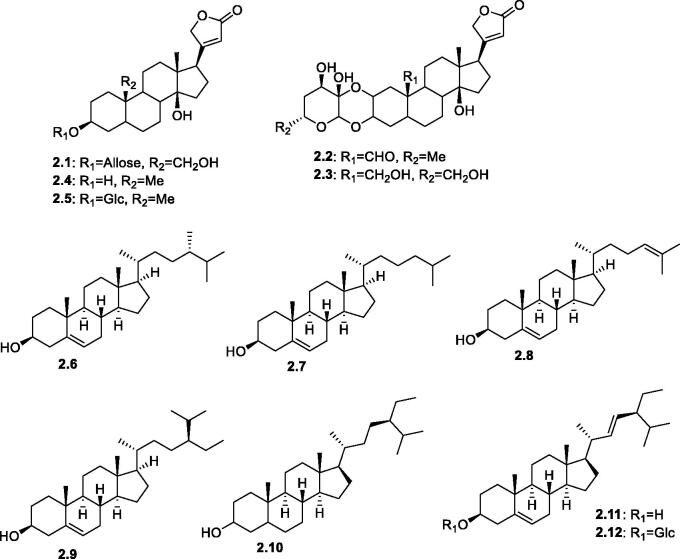
Structures of cardenolides and steroids (**2.1–2.12**) reported in the genus *Salsola.*

Steroids are a group of natural products biosynthesized from the isoprenoid pathway via the 2,3-oxidosqualene (C_30_) route. Cardenolides are cardioactive steroidal lactones with a 5-membered (furanones) or 6-membered (pyranone) ring at C-17. They are naturally present free or glycosylated with mono- or multi-sugar moieties. Several families are known for their high cardenolides content, such as Asclepidaceae, Apocynaceae, and others^72^. However, only one report on cardenolides from the Amaranthaceae family has been described. It addressed the isolation of five cardenolides, salsotetragonin **2.1**, calactin **2.2**, 12-dehydroxyghalakinoside **2.3**, desglucouzarin **2.4**, and uzarigenin **2.5** from the Algerian plant, *Salsola tetragona* Delile, Figure 3^50^. Other reported steroids comprised several phytosterols with diversity in the alkyl side chains at C-17, including campesterol **2.6**, cholesterol **2.7**, and desmosterol **2.8** from *S. collina*^73^, β-sitosterol **2.9**, stigmastanol **2.10**, and stigmasterol **2.11**, in addition to a combined phytosterol, stigmasterol-3-*O*-β-D-glucopyranoside **2.12** from the aerial parts of *S. inermis*^51^.

The existence of fatty acid esters or acylated sterols was reported by Mayakova et al.^73^ from the genus *Salsola*. They investigated the contents of the saponified acylsterols fraction of the pentane extract of *S. collina*. The neutral fraction indicated the presence of four sterols, including β-sitosterol, stigmasterol, cholesterol, and campesterol, whereas the acyl fraction of the hydrolysed esters composed of stearic, palmitic, and oleic acids^73^.

#### 4.1.4. Coumarins and coumarinolignans

**Figure 4.**
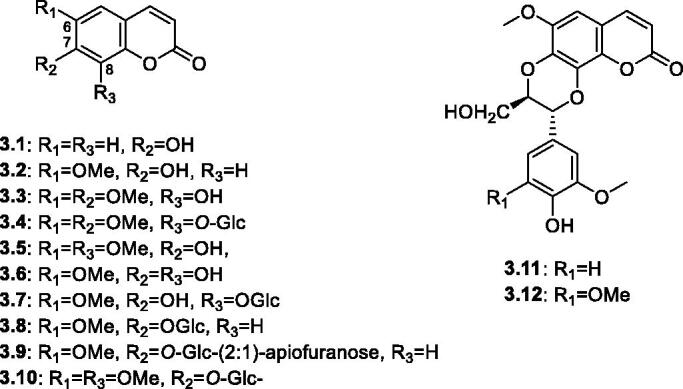
Structures of coumarins and coumarinolignans (**3.1–3.12**) reported in the genus *Salsola.*

Coumarins are bioactive secondary metabolites biosynthesized in plants from the phenylpropanoid (C_6_C_3_) pathway by cyclisation of cinnamic acid. They contribute to diverse biological activities, such as anticoagulant, antimicrobial, antiviral, and anticancer activities^74^. Several studies reported the presence of simple coumarins in members of the genus *Salsola*. These reported coumarins are either free or glycosylated with mostly methoxylated C-6 and oxygenated C-7 positions. Two simple coumarins, namely umbelliferone **3.1** and scopoletin **3.2** were reported from the aerial parts of *S. inermis*^51^. Whereas *S. kali* showed the presence of fraxidin **3**.**3^75^** . However, the highest record of coumarins from this genus was noted to *S. laricifolia* that included several simple coumarins (**3.3**–**3.10**) and two unusual coumarinolignans; cleomiscosin B **3.11,** cleomiscosin D **3.12**, formed by the association with another cinnamic acid moiety (C_6_C_3_)^52^. Calycantoside **3.10**, a compound possessing the structure of 6,8-dimethoxy-coumarin-7-*O*-*β*-glucopyranoside was reported with the miss-spelled name, calicantoside from the epigeal (aerial) parts of *S. laricifolia*^76^ Figure 4.

#### 4.1.5. Fatty acids and their derivatives

**Figure 5.**
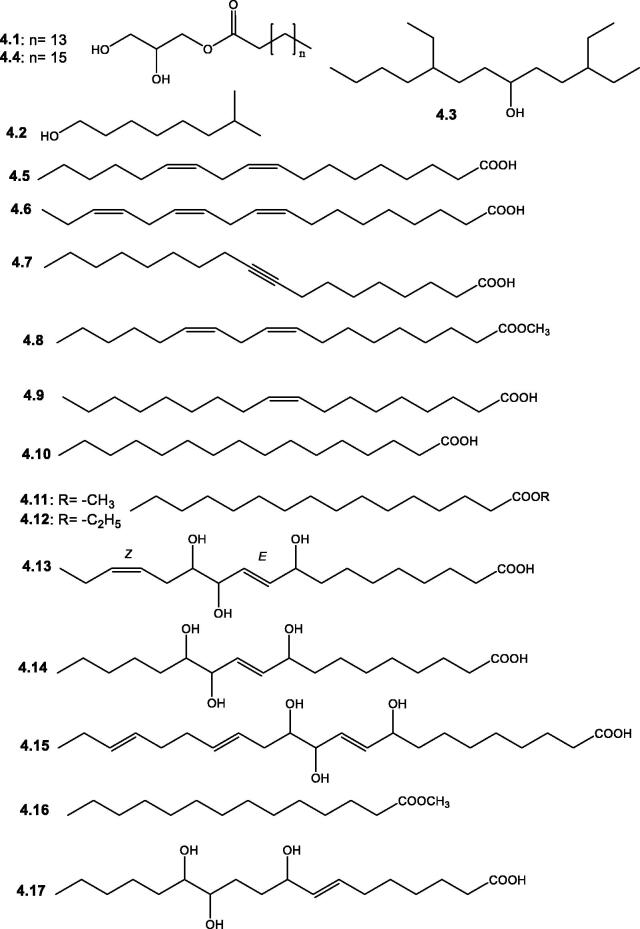
Structures of fatty acids and their derivatives (**4.1**–**4.17**) reported in the genus *Salsola.*

Few saturated fatty acids compared to unsaturated ones were reported from *Salsola* plants, Table 2 and Figure 5. Ghorab et al.^50^ reported the isolation of the fatty acid ester, 2,3-dihydroxypropylpalmitate **4.1** from the aerial parts of *S. tetragona*. Whereas free palmitic acid **4.10**, in addition to three unsaturated fatty acids, including linoleic, linolenic, and oleic acids (**4.5**, **4.6**, and **4.9**, respectively) were detected by UPLC/qTOF-MS analysis of *S. vermiculata* and *S. tetrandra*^68^. Also, oleic acid **4.9** was isolated from the aerial parts of *S. tetragona*^50^. A characteristic group of trihydroxylated mono-, di-, and tri-unsaturated fatty acids was reported from several plants of the genus *Salsola*, including 9,12,13-trihydroxyoctadeca-10(*E*),15(*Z*)-dienoic acid **4.13** and 9,12,13-trihydroxy-10(*E*)-octadecenoic acid **4.14** from the aerial parts of *S. tetrandra*^53^ and 9,12,13-trihydroxydocosan-10,15,19-trienoic acid **4.15** from the aerial parts of *S. inermis*^51^. Additionally, several fatty acids, including hydroxyoctadecenoic acid, dihydroxyoctadecenoic acid, hydroxyoctadecatrienoic acid, hydroxyoctadecadienoic acid, and trihydroxyoctadecadienoic acid were also tentatively identified from the aerial parts and roots of *S. vermiculata* and *S. tetrandra* by UPLC/qTOF-MS analysis method^68^.

**Table 2.** Non-volatile constituents from the genus *Salsola.*

No.	Class/Name	Plant/ part	Reference
**I- Alkaloids and nitrogenous compounds**			
**1.1**	*N*-Acetyltryptophan	The whole plant of *S. collina* Pall.; *S. grandis* Freitag, Vural & Adiguzel	^66^ ^,^ ^70^ ^,^ ^77^
**1.2**	Carnegine	*S. richteri*; GC/MS of the aerial parts of *S. oppositifolia* Desf.	^45^ ^,^ ^48^
**1.3**	*N*-[2-(3,4-Dihydroxyphenyl)-2-hydroxyethyl]-3-(4-methoxyphenyl)prop-2-enamide	The whole plant of *S. foetida*	^78^
**1.4**	*N*-[2-(3,4-Dihydroxyphenyl)-2-hydroxyethyl]-3-(3,4-dimethoxyphenyl)prop-2-enamide	The whole plant of *S. foetida*	^78^
**1.5**	*Cis*-*N*-Feruloyltyramine	The aerial parts of *S. baryosoma*	^67^
**1.6**	*N*-*Trans*-feruloyl-3′″-methoxydopamine 4′-*O*-β-D-glucopyranoside	The aerial parts of *S. collina*	^43^
**1.7**	*Trans*-*N*-Feruloyl tyramine-4′″-*O*-β-D-glucopyranoside	The aerial parts of *S. inermis* Forssk	^51^
**1.8**	*N*-[2-(3-Hydroxy-4-methoxyphenyl)-2-hydroxyethyl]3-(4-methoxyphenyl)-prop-2-enamide	The whole plant of *S. foetida*	^78^
**1.9**	7′-Hydroxy-3′-methylmoupinamide; *N*-*trans*-feruloyl-3-*O*-methyldopamine	The whole plant of *S. collina* Pall.; HPLC of the aerial parts of *S. komarovii*	^43^ ^,^ ^66^ ^,^ ^79^
**1.10**	7′-Hydroxymoupinamide (7′-Hydroxy *N*-*trans*- feruloyltyramine); *trans*-*N*-Feruloyloctopamine	The whole plant of *S. collina* Pall. and aerial parts of *S. tetrandra;* aerial parts of *S. baryosoma*	^53^ ^,^ ^66^ ^,^ ^67^
**1.11**	Methyl carbamate	*S. tetrandra*, *S. kali*, *S. longifolia* and *S. rigida*	^69^
**1.12**	*N*-Methylisosalsoline	By GC/MS of the aerial parts of *S. tragus* L., *S. oppositifolia* Desf., and *S. soda* L.	^48^
**1.13**	Moupinamide (*N*-*trans*-Feruloyltyramine)	The whole plant of *S. collina* Pall. and aerial parts of *S. tetrandra*; UPLC/qTOF-MS analysis of whole plants of *S. vermiculata* and *S. Tetrandra*; Forssk; aerial parts of *S. baryosoma*; HPLC of the aerial parts of *S. komarovii*	^43^ ^,^ ^53^ ^,^ ^66–68^ ^,^ ^80^ ^,^ ^79^
**1.14**	Pericampylinone-A (iseluxine)	The whole plant of *S. collina* Pall.	^66^
**1.15**	Salisomide	The arial parts *S. imbricata* Forssk	^57^
**1.16**	Salsoline	Aerial parts and root of *Salsola kali* L. and *S. longifolia* Forssk; GC/MS of the aerial parts of *S. tragus* L., *S. oppositifolia* Desf., and *S. soda* L.	^44^ ^,^ ^45^ ^,^ ^48^ ^,^ ^75^ ^,^ ^81^
**1.17**	Salsoline A (Trolline)	*S. collina* Pall.; UPLC/qTOF-MS analysis of whole plants of *S. vermiculata* and *S. tetrandra*	^47^ ^,^ ^49^ ^,^ ^66^ ^,^ ^68^
**1.18**	Salsoline B	*S. collina* Pall.	^43^
**1.19**	Salsolidine (*N*-Norcarnegine)	The aerial parts of *S. kali* L. and *S. longifolia* Forssk; GC/MS of the aerial parts of *S. tragus* L., *S. oppositifolia* Desf., and S. soda L.	^45^ ^,^ ^48^ ^,^ ^81^
**1.20**	Terrestric acid; 4-Amino-1,2,5,6-tetrahydro-6-oxo-1,3,5-triazine-2-carboxylic acid	The whole plant of *S. collina* Pall.	^66^
**1.21**	Tridecanamine	By GC-MS analysis of the aerial parts of *S. tetrandra*	^71^
**1.22**	Uracil	The whole plant of *S. collina* Pall.	^66^
**1.23**	Uridine	The whole plant of *S. collina* Pall.	^66^
**II- Cardenolides and steroids**			
**A. Cardenolides**			
** 2.1**	3-*O*-β-D-Allopyranosylcoroglaucigenin (salsotetragonin)	The aerial parts of *S. tetragona*	^50^
** 2.2**	Calactin	The aerial parts of *S. tetragona*	^50^
** 2.3**	12-Dehydroxyghalakinoside	The aerial parts of *S. tetragona*	^50^
** 2.4**	Desglucouzarin	The aerial parts of *S. tetragona*	^50^
** 2.5**	Uzarigenin	The aerial parts of *S. tetragona*	^50^
**B. Steroids**			
** 2.6**	Campesterol	*S. collina*	^73^
** 2.7**	Cholesterol	*S. collina*	^73^
** 2.8**	Desmosterol	*S. collina*	^73^
** 2.9**	*β*-Sitosterol	The aerial parts of *S. inermis*; *S. collina*	^51^ ^,^ ^73^
** 2.10**	Stigmastanol	The aerial parts of *S. inermis*	^51^
** 2.11**	Stigmasterol	The aerial parts of *S. inermis*; *S. collina*	^51^ ^,^ ^73^
** 2.12**	Stigmasterol-3-*O*-β-D-glucopyranoside	The aerial parts of *S. inermis*	^51^
**III- Coumarins and coumarinolignans**			
**3.1**	Umbelliferone	The aerial parts of *S. inermis*	^51^
**3.2**	Scopoletin	The aerial parts of *S. inermis*	^51^
**3.3**	Fraxidin	The epigeal part of *S. laricifolia*; Herb and root of *S. kali* L.	^75^ ^,^ ^82^
**3.4**	Fraxidin-8-*O*-β-D-glucopyranoside	The epigeal part of *S. laricifolia*	^82^
**3.5**	Isofraxidin	The epigeal part of *S. laricifolia*	^82^
**3.6**	Fraxetin	The epigeal part of *S. laricifolia*	^82^
**3.7**	Fraxin	The epigeal part of *S. laricifolia*	^82^
**3.8**	Scopolin	The epigeal part of *S. laricifolia*	^82^
**3.9**	7-[*O*-β-D-Apiofuranosyl-(l→ 2)-6-D-glucopyranosyloxy]- 6-methoxy-2*H*-l-benzopyran-2-one (lariside)	The epigeal part of *S. laricifolia*	^82^ ^,^ ^83^
**3.10**	Calycantoside; Calicantoside	The epigeal part of *S. laricifolia*	^76^ ^,^ ^82^
**3.11**	Cleomiscosin B	*S. laricifolia*	^52^
**3.12**	Cleomiscosin D	*S. laricifolia*	^52^
**IV- Fatty acids and their derivatives**			
**4.1**	2,3-Dihydroxypropylpalmitate	The aerial parts of *S. tetragona*	^50^
**4.2**	2,7-Dimethyl-1-octanol	By GC-MS analysis of the aerial parts of *S. tetrandra*	^71^
**4.3**	3,9-Diethyl-6-tridecanol	By GC-MS analysis of the aerial parts of *S. tetrandra*	^71^
**4.4**	2,3-Dihydroxypropyl octadecanoate	By GC-MS analysis of the aerial parts of *S. tetrandra*	^71^
**4.5**	Linoleic acid	UPLC/qTOF-MS analysis of whole plants of *S. vermiculata* and *S. tetrandra*	^68^
**4.6**	Linolenic acid	UPLC/qTOF-MS analysis of whole plants of *S. vermiculata* and *S. tetrandra*	^68^
**4.7**	9-Octadecynoic acid	By GC-MS analysis of the aerial parts of *S. tetrandra*	^71^
**4.8**	9,12-Octadecadienoic (*Z*,*Z*) methyl ester	By GC-MS analysis of the aerial parts of *S. tetrandra*	^71^
**4.9**	Oleic acid	The aerial parts of *S. tetragona*; UPLC/qTOF-MS analysis of whole plants of *S. vermiculata* and *S. tetrandra*	^50^ ^,^ ^68^
**4.10**	Palmitic acid; Hexadecenoic acid	UPLC/qTOF-MS analysis of whole plants of *S. vermiculata* and *S. tetrandra*	^68^
**4.11**	Palmitic acid methyl ester; methyl palmitate	By GC-MS analysis of the aerial parts of *S. tetrandra*	^71^
**4.12**	Palmitic acid ethyl ester; Hexadecenoic acid ethyl ester	By GC-MS analysis of the aerial parts of *S. tetrandra*	^71^
**4.13**	9,12,13-Trihydroxyoctadeca-10(*E*),15(*Z*)-dienoic acid	The aerial parts of *S. tetrandra*	^53^
**4.14**	9,12,13-Trihydroxy-10(*E*)-octadecenoic acid		
**4.15**	9,12,13-Trihydroxydocosan-10,15,19-trienoic acid	The aerial parts of *S. inermis*	^51^
**4.16**	Tetradecanoic acid methyl ester	By GC-MS analysis of the aerial parts of *S. tetrandra*	^71^
**4.17**	9,​12,​13-​Trihydroxy-​7-​octadecenoic acid	UPLC/qTOF-MS analysis of whole plants of *S. vermiculata* and *S. tetrandra*	^68^
**V- Flavonoids and flavonolignans:**			
**A. Flavones and their derivatives**			
** 5.1**	Apigenin	HPLC analysis of whole plant of *S. imbricata* Forssk	^84^
** 5.2**	Chrysin	HPLC analysis of whole plant of *S. imbricata* Forssk	^84^
** 5.3**	Flavonol (Flavon-3-ol; 3-Hydroxyflavone)	*S. grandis* Freitag, Vural & Adiguzel	^70^
** 5.4**	Isorhamnetin	The whole plant of *S. collina* Pall.; leaves of *S. Imbricata*; HPLC of the aerial parts of *S. komarovii*	^66^ ^,^ ^79^ ^,^ ^80^
** 5.5**	Isorhamnetin-3-*O*-rutinoside (Narcissoside)	*S. kali*; UPLC/qTOF-MS analysis of whole plants of *S. vermiculata* and *S. tetrandra*; *S. grandis* Freitag, Vural & Adiguzel; aerial parts of wild *S. soda*; aerial parts of *S. Oppositifolia*; HPLC of the aerial parts of *S. komarovii*	^10^ ^,^ ^54^ ^,^ ^68^ ^,^ ^70^ ^,^ ^77^ ^,^ ^79^ ^,^ ^85^
** 5.6**	Isorhamnetin-3-*O*-α-L-arabinopyranosyl (1→6)-β-D-glucopyranoside	The whole plant of *S. collina*	^60^
** 5.7**	Isorhamnetin-3-*O*-β-D-galactopyranoside	*S. grandis* Freitag, Vural & Adiguzel Leaves of *S. Imbricata*	^70^ ^,^ ^77^ ^,^ ^80^
** 5.8**	Isorhamnetin-3-*O*-β-D-glucopyranoside	The whole plant of *S. collina*; aerial parts of *S. inermis,* and *S. kali*; UPLC/qTOF-MS analysis of whole plants of *S. vermiculata* and *S. tetrandra*; *S. grandis* Freitag, Vural & Adiguzel; leaves of *S. imbricata* Forssk; aerial parts of *S. oppositifolia*; HPLC of the aerial parts of *S. komarovii*	^51,54,60,68,70,77^,^79^,^80^,^85^
** 5.9**	Isorhamnetin-7-*O*-β-D-glucopyranoside	The whole plant of *S. collina*	^60^
** 5.10**	Isorhamnetin-3-*O*-*β*-D-glucuronate methyl ester (1′″→4′`)-β-glucuronate methyl ester	Leaves of *S. imbricata* Forssk	^80^
** 5.11**	Isorhamnetin-3-*O*-*β*-D-glucuronide	*S. grandis* Freitag, Vural & Adiguzel; aerial parts of wild *S. soda*	^10^ ^,^ ^70^ ^,^ ^77^
** 5.12**	Isorhamnetin-3-*O*-*β*-D-glucuronyl-(1′``→4′`)-β-D-glucuronic acid	Leaves of *S. imbricata* Forssk	^80^
** 5.13**	Kaempferol-3-*O*-methylether	The aerial parts of *S. inermis*	^51^
** 5.14**	Kaempferol-3-*O*-β-D-(6′`-*O*-(*E*)-p-coumaroyl)glucopyranoside); *trans*-Tiliroside	*S. grandis* Freitag, Vural & Adiguzel	^70^ ^,^ ^77^
** 5.15**	Kaempferol-3-*O*-β-D-glucopyranoside; Astragalin	The aerial parts of *S. tetragona,* and *S. inermis*; HPLC of the aerial parts of *S. komarovii*	^50^ ^,^ ^51^ ^,^ ^79^
** 5.16**	Kaempferol-3-*O*-rutinoside	HPLC of the aerial parts of *S. komarovii*	^79^
** 5.17**	Luteolin-7-*O*-β-D-glucoside	HPLC analysis of aerial parts and root of *S. kali* L.	^75^
** 5.18**	Quercetin	*S. collina* Pall*., S. kali*; HPLC analysis of *S. imbricata* Forssk; UPLC/qTOF-MS analysis of whole plants of *S. vermiculata* and *S. tetrandra*; *S. grandis* Freitag, Vural & Adiguzel; LC-MS of *S. cyclophylla*	^15^ ^,^ ^66^ ^,^ ^68^ ^,^ ^70^ ^,^ ^77^ ^,^ ^84^
** 5.19**	Quercetin-3-*O*-β-D-galactoside; Hyperin; Hyperoside	HPLC analysis of aerial parts and root of *S. kali* L.; *S. grandis* Freitag, Vural & Adiguzel; LC-MS analysis of *S. cyclophylla*	^15^ ^,^ ^70^ ^,^ ^77^ ^,^ ^75^
** 5.20**	Quercetin-3-​*O*-​glucopyranoside; Isoquercitrin	HPLC of the aerial parts of *S. komarovii*	^79^
** 5.21**	Quercetin-3-*O*-β-D-glucopyranosyl-(1→6)-glucopyranoside	The aerial parts of *S. tetragona*	^50^
** 5.22**	Quercetin-3-​*O*-​glucuronopyranoside	The aerial parts of wild *S. soda*	^10^ ^,^ ^79^
** 5.23**	Quercetin-3-*O*-methylether	*S. grandis* Freitag, Vural & Adiguzel	^77^
** 5.24**	Quercetin 3-α-L-rhamnoside; Quercetrin	HPLC analysis of whole plant of *S. imbricata* Forssk; *S. grandis* Freitag, Vural & Adiguzel	^70^ ^,^ ^77^ ^,^ ^84^
** 5.25**	Quercetin-3-*O*-rutinoside; Rutin	*S. collina* Pall.; HPLC analysis of *S. imbricata* Forssk; UPLC/qTOF-MS analysis of whole plants of *S. vermiculata* and *S. tetrandra*; *S. grandis* Freitag, Vural & Adiguzel; aerial parts of wild *S. soda*; HPLC of the aerial parts of *S. komarovii*	^10^ ^,^ ^66^ ^,^ ^68^ ^,^ ^70^ ^,^ ^77^ ^,^ ^79^ ^,^ ^84^
** 5.26**	Quercetin 3-*O*-rutinoside-(1:2)-*O*-rhamnoside; Quercetin 3-*O*-(2′`,6′`-di-*O*-*α*-L-rhamnopyranosyl)-*β*-D-glucopyranoside (Manghaslin)	*S. grandis* Freitag, Vural & Adiguzel	^70^ ^,^ ^77^
** 5.27**	Selagin; 3′-*O*-Methyltricetin	The whole plant of *S. collina Pall.*	^60^
** 5.28**	Tricin	The whole plant of *S. collina* Pall.	^60^ ^,^ ^66^
** 5.29**	Tricin*-*4′-*O*-[*erythro*-β-guaiacylglyceryl] ether; *Erythro*-4′-*O*-(β-guaiacylglyceryl)tricin (salcolin B)	The epigeal part of *S. collina*	^56^ ^,^ ^86^
** 5.30**	Tricin*-*4′-*O*-[*threo*-β-guaiacylglyceryl] ether; *Threo*-4′-*O*-(β-guaiacylglyceryl)tricin (salcolin A)	The epigeal part of *S. collina*	
** 5.31**	Tricin-7-*O*-β-D-glucopyranoside	The whole plant of *S. collina* Pall	^60^ ^,^ ^66^
** 5.32**	Tricin-4′-*O*-β-D-apioside	The whole plant of *S. collina*	^60^
**B. Flavanols and flavanones**			
** 5.33**	Catechin	HPLC analysis of whole plant of *S. imbricata* Forssk	^84^
** 5.34**	Hesperidin	HPLC analysis of whole plant of *S. imbricata* Forssk	^84^
** 5.35**	Hesperitin	HPLC analysis of whole plant of *S. imbricata* Forssk	^84^
** 5.36**	Naringenin	HPLC analysis of whole plant of *S. imbricata* Forssk	^84^
**C. Isoflavonoids**			
** 5.37**	5,2′-Dihydroxy-5′-methoxy-6,7-methylenedioxy-isoflavone (Tetranin B)	*S. tetrandra* Folsk roots	^59^
** 5.38**	5,2′-Dihydroxy-6,7-methylenedioxyisoflavone (Irisone B)	The whole plant of *S. collina* Pall.	^66^ ^,^ ^87^
** 5.39**	5,3′-Dihydroxy-2′-methoxy-6,7-methylenedioxyisoflavone	The roots of *S. somalensis*	^55^ ^,^ ^88^
** 5.40**	5,3′-Dihydroxy-6,7,2′-trimethoxyisoflavone	The roots of *S. somalensis*	^55^
** 5.41**	5,3′-Dihydroxy-6,7,8,2′-tetramethoxyisoflavone	The roots of *S. somalensis*	^55^ ^,^ ^88^
** 5.42**	5,3′-Dihydroxy-7,8,2′-trimethoxyisoflavone	The roots of *S. somalensis*	^55^ ^,^ ^88^
** 5.43**	6,3′-Dihydroxy-5,7,2′-trimethoxyisoflavone	The roots of *S. somalensis*	^55^
** 5.44**	7,3′-Dihydroxy-5,6,2′-trimethoxyisoflavone	The roots of *S. somalensis*	^55^
** 5.45**	8,3′-Dihydroxy-5,7,2′-trimethoxyisoflavone	The roots of *S. somalensis*	^55^
** 5.46**	3′-Hydroxy-5,6,7,2′-tetramethoxyisoflavone	The roots of *S. somalensis*	^55^
** 5.47**	5,6,3′-Trihydroxy-7,2′-dimethoxyisoflavone	The roots of *S. somalensis*	^55^
** 5.48**	5,8,3′-Trihydroxy-7,2′-dimethoxyisoflavone	The roots of *S. somalensis*	^55^
** 5.49**	6,7,3′-Trihydroxy-5,2′-dimethoxyisoflavone	The roots of *S. somalensis*	^55^
** 5.50**	5,2′,3′-Trimethoxy-6,7- methylenedioxyisoflavone	The roots of *S. somalensis*	^88^
** 5.51**	5,6,7,2′,3′-Pentamethoxyisoflavone	The roots of *S. somalensis*	^55^
** 5.52**	5,7,8,2′,3′-Pentamethoxyisoflavone	The roots of *S. somalensis*	^88^
**D. Isoflavan**			
** 5.53**	Salisoflavan	The arial parts *S. imbricata* Forssk	^57^
**VI-Lignans**			
**6.1**	Acanthoside D	The whole plant of *S. collina*	^60^
**6.2**	Alangilignoside C	The aerial parts of *S. komarovii*	^89^
**6.3**	Conicaoside	The aerial parts of *S. komarovii*	^89^
**6.4**	(8*S*,8′*R*,7′*R*)-9′-[(*β*-Glucopyranosyl)oxy]lyoniresinol	The aerial parts of *S. komarovii*	^89^
**6.5**	Lariciresinol-9-*O*-β-D-glucopyranoside	The aerial parts of *S. komarovii*	^89^
**6.6**	(+)-Lyoniresinol 9′-*O*-*β*-D-glucopyranoside	The aerial parts of *S. komarovii*	^89^
**VII- Triterpenoids and their derivatives**			
**A. Triterpenoids**			
** 7.1**	3-*O*-*β*-D-Glucopyranosyl-6*β*,11*β*,23,24- tetrahydroxyolean-12-en-28-oic acid	The whole plant of *S. baryosma*	^64^
** 7.2**	Guavenoic acid; 2α,3β,6β,23-Tetrahydroxyursa-12,20(30)-dien-28-oic acid	*S. baryosma*	^90^
** 7.3**	Momordin IIb; Silphioside G; Oleanolic acid 3-glucuronide 28-glucoside	*S. imbricata* Forssk root; *S. grandis* Freitag, Vural & Adiguzel	^61^ ^,^ ^70^ ^,^ ^77^
** 7.4**	Momordin IId; 3β-(([*O*-β-D-Xylopyranosyl-(1→2)-*O*-β-D-xylopyranosyl-(1→3)]-*O*-β-D-glucopyranuronosyl)oxy)-olean-12-ene-28-glucopyranoside	By HPLC-ESI-MS from aerial parts of wild *S. soda*	^10^
** 7.5**	Olean-12-en-3,28-diol	The aerial parts of *S. inermis*	^51^
** 7.6**	Oleanolic acid; Olean-12-en-28-oic acid	The aerial parts of *S. inermis*; aerial parts of wild *S. soda*	^10^ ^,^ ^51^
** 7.7**	Oleanolic acid-3-*O*-β-D-glucopyranosyl	The aerial parts of *S. inermis*	^51^
** 7.8**	1α,2α,3β,19α,23-Pentahydroxyursa-12,20(30)-dien-28-oic acid	*S. baryosma*	^90^
** 7.9**	Pseudoginsenoside RT1	*S. imbricata* Forssk root	^61^
** 7.10**	Salsolin A; 3*β*,11*β*,24,30-Tetrahydroxyolean-12-en-28-oic acid	The whole plant of *S. baryosma*	^64^
** 7.11**	Salsolin B; 2α,3β,23,24-Tetrahydroxyurs-12-en-28-oic acid	The whole plant of *S. baryosma*	^64^
** 7.12**	Salsolic acid; 3β,6α,24-Trihydroxyolean-12-en-28-oic acid	*S. baryosma*	^90^
** 7.13**	Salsoloside C; Momordin IIc; Oleanolic acid 28-*O*-β-D-glucopyranoside 3-*O*-[*O*-β-D-xylopyranosyl-(l→4)-β-*D*-glucuropyranoside]	The epigeal part of *S. micranthera* Botsch; *S. grandis* Freitag, Vural & Adiguzel; By HPLC-ESI-MS aerial parts of wild *S. soda*	^10^ ^,^ ^62^ ^,^ ^70^ ^,^ ^77^
** 7.14**	Salsoloside D; Hederagenin 28-*O*-β-D-glucopyranoside 3-*O*-[*O*-β-D-xylopyranosyl-(l→4)-β-D-glucuropyranoside]	The epigeal part of *S. micranthera* Botsch	^62^
** 7.15**	Salsoloside E; Oleanolic acid 28-*O*-β-D-glucopyranoside 3-*O*-[*O*-β-D-glucopyranosyl-(1→2)-[*O*-β-D-xylopyranosyl-(l→4)-β-D-glucuropyranoside]	The epigeal part of *S. micranthera* Botsch	^63^
** 7.16**	3-*O*-β-D-Xylopyranosyl-(1→2)- *O*-β-D-glucuronopyranosyl-29-hydroxyoleanolic acid 28-*O*-β-D-glucopyranoside	*S. imbricata* Forssk root	^61^
**B. Nortriterpenoids**			
** 7.17**	3-*O*-β-D-Glucuronopyranosyl-30-norolean-12,20(dien-28-*O*-[β-D-glucopyranosyl] ester (boussingoside A2)	*S. imbricata* Forssk root	^61^
** 7.18**	3-*O*-β-D-Xylopyranosyl-(1→2)-*O*-β-D-glucuronopyranosyl-akebonic acid 28-*O*-β-D-glucopyranoside	*S. imbricata* Forssk root	^61^
**VIII- Phenolic acids and simple phenols**			
**8.1**	Acetyl ferulic acid	*S. collina* Pall.	^66^
**8.2**	Anisic acid	*S. collina* Pall.	^66^
**8.3**	Benzoic acid	HPLC analysis of whole plant of *S. imbricata* Forssk	^84^
**8.4**	Caffeic acid	HPLC analysis of whole plants of *S. kali* and *S. imbricata* Forssk; UPLC/qTOF-MS analysis of whole plants of *S. vermiculata* and *S. tetrandra*	^12^ ^,^ ^68^ ^,^ ^84^
**8.5**	Caffeic acid phenethyl ester; β-​Phenylethyl caffeate	LC-MS analysis of *S. cyclophylla*	^15^
**8.6**	Catechol	HPLC analysis of herb and root of *S. kali*	^12^
**8.7**	Chlorogenic acid	HPLC analysis of whole plant of *S. imbricata* Forssk; LC-MS analysis of *S. cyclophylla*	^15^ ^,^ ^84^
**8.8**	Cinnamic acid	HPLC analysis of whole plant of *S. imbricata* Forssk; LC-MS analysis of *S. cyclophylla*	^15^ ^,^ ^84^
**8.9**	*p*-Coumaric acid	HPLC analysis of whole plants of *S. kali* and *S. imbricata* Forssk; *S. collina* Pall.; LC-MS analysis of *S. cyclophylla*	^12^ ^,^ ^15^ ^,^ ^60^ ^,^ ^66^ ^,^ ^84^
**8.10**	Ferulic acid	Whole plant of *S. collina*; HPLC analysis of whole plants of *S. kali* and *S. imbricata* Forssk; UPLC/qTOF-MS analysis of whole plants of *S. vermiculata* and *S. Tetrandra;* LC-MS analysis of *S. cyclophylla*	^12^ ^,^ ^15^ ^,^ ^60^ ^,^ ^68^ ^,^ ^80^ ^,^ ^84^
**8.11**	Gallic acid	HPLC analysis of whole plant of *S. imbricata* Forssk; LC-MS of *S. cyclophylla*	^15^ ^,^ ^84^
**8.12**	Gentisic acid	HPLC analysis of herb and root of *S. kali*	^12^
**8.13**	4-Hydroxy-acetophenone; 1-(4-hydroxy-phenyl)-ethanone	*S. tuberculatiformis* Botsch.	^40^
**8.14**	4-Hydroxy-3-methoxy-acetophenone; 1-(4-hydroxy-3-methoxy-phenyl)-ethanone	*S. tuberculatiformis* Botsch.	^40^
**8.15**	4-Hydroxybenzaldehyde	*S. tuberculatiformis* Botsch.	^40^
**8.16**	*p*-Hydroxybenzoic acid	*S. collina* Pall.; HPLC analysis of herb and root of *S. kali*; leaves of and *S. imbricata* Forssk	^12^ ^,^ ^66^ ^,^ ^80^
**8.17**	*p*-Hydroxyphenylacetic acid	HPLC analysis of herb and root of *S. kali*	^12^
**8.18**	Isovanillic acid	Leaves of *S. imbricata* Forssk	^80^
**8.19**	Protocatechuic aldehyde	*S. collina* Pall.	^66^
**8.20**	Protocatechuic acid	HPLC analysis of whole plants of *S. kali* and *S. imbricata* Forssk	^12^ ^,^ ^84^
**8.21**	Resorcinol	HPLC analysis of aerial parts and root of *S. kali* L.	^75^
**8.22**	*α*-Resorcylic acid	HPLC analysis of herb and root of *S. kali*	^12^
**8.23**	*β*-Resorcylic acid	HPLC analysis of herb and root of *S. kali*	^12^
**8.24**	Rosmarinic acid	HPLC analysis of *S. imbricata* Forssk	^84^
**8.25**	Salicylic acid	*S. collina* Pall.; HPLC analysis of whole plants of *S. imbricata* Forssk	^60^ ^,^ ^66^ ^,^ ^84^
**8.26**	Syringic acid	HPLC analysis of herb and root of *S. kali*	^12^
**8.27**	Tetranin A	*S. tetrandra* Folsk roots	^59^
**8.28**	Vanillic acid	HPLC analysis of whole plants of *S. kali* and *S. imbricata* Forssk; from the aerial parts of *S. tetragona*	^12^ ^,^ ^50^ ^,^ ^84^
**8.29**	Vanillin	*S. collina* Pall.	^66^
**IX- Miscellaneous glycosides**			
**9.1**	Benzyl 6-*O*-*β*-D-apiofuranosyl-*β*-D-glucopyranoside	The aerial parts of *S. komarovii*	^89^
**9.2**	Biophenol 2	The aerial parts of *S. komarovii*	^89^
**9.3**	Blumenol B 9-*O*-*β*-D-apiofuranosyl-(1→6)-β-D-glucopyranoside	The aerial parts of *S. komarovii*	^89^
**9.4**	Blumenyl A *β*-D-glucopyranoside; Roseoside A	The aerial parts of *S. komarovii*	^89^
**9.5**	Blumenyl B *β*-D-glucopyranoside	The aerial parts of *S. komarovii*	^89^
**9.6**	Canthoside C	The aerial parts of *S. tetragona* and *S. komarovii*	^50^ ^,^ ^89^
**9.7**	Canthoside D	The aerial parts of *S. tetragona*	^50^
**9.8**	Corchoionoside C	The whole plant of *S. collina* Pall.	^66^
**9.9**	Cuneataside C	The aerial parts of *S. komarovii*	^89^
**9.10**	2-(3,4-Dihydroxy)-phenyl-ethyl-β-D-glucopyranoside	The aerial parts of *S. komarovii*	^89^
**9.11**	9-​Hydroxylinaloyl glucoside	The aerial parts of *S. tetrandra*	^53^
**9.12**	Icariside B2	The aerial parts of *S. komarovii*	^89^
**9.13**	Isotachioside	The aerial parts of *S. komarovii*	^89^
**9.14**	Lyohebecarpin A (3*β*-Hydroxy-5*R*,6*R*-epoxy-*β* -ionone-2*R*-*O*-*β*-D-glucopyranoside)	The aerial parts of *S. tetrandra*	^53^
**9.15**	Staphylionoside D	The aerial parts of *S. komarovii*	^89^
**9.16**	Tachioside	The aerial parts of *S. komarovii*	^89^
**9.17**	Taxiphyllin	The aerial parts of *S. tetrandra*	^53^
**9.18**	3,4,5-Trimethoxyphenyl-*β*-D-glucopyranoside	The aerial parts of *S. tetrandra*	^53^
**9.19**	(6*R*,9*S*)-3-Oxo-*α*-ionol *β*-D-glucopyranoside	The aerial parts of *S. komarovii*	^89^
**9.20**	3-Oxo-*α*-ionol 9-*O*-*β*-D-apiofuranosyl-(1→6)-*β*-D-glucopyranoside	The aerial parts of *S. komarovii*	^89^
**X- Phenylpropanoids**			
**10.1**	Biphenylsalsinol; 4′-[3-(hydroxymethyl)oxiran-2-yl]-3- [(*E*)-3-hydroxyprop-1-en-1-yl]-6, 2′-dimethoxy [1, 1′-biphenyl]-2-ol	The aerial parts of *S. villosa* Delile. ex Schul.	^91^
**10.2**	Biphenylsalsonoid A; 4′-(9′- (Hydroxymethyl) oxiran-7′-yl)-4-((E)-3-hydroxyprop-7-en-7-yl)-3,3′-dimethoxy-[1,1′-biphenyl]-2,5′-diol	Roots of *S. imbricata*	^92^
**10.3**	Biphenylsalsonoid B; 4,4′-bis-(9-hydroxymethyl) oxiran-7-yl)-5,3′,5′-trimethoxy [1,1′biphenyl]-3-ol	Roots of *S. imbricata*	^92^
**XI- Polyhydric alcohols and carbohydrates**			
**11.1**	Ethyl β-D-fructopyranoside	*S. collina* Pall.	^93^
**11.2**	Ethyl β-D-glucopyranoside	*S. collina* Pall.	^93^
**11.3**	D-Fructose	*S. collina* Pall.	^93^
**11.4**	D-Glucose	*S. collina* Pall.	^93^
**11.5**	D-Mannitol	*S. collina* Pall.	^93^
**11.6**	Myoinositol	*S. collina* Pall.	^93^
**XII- Miscellaneous group**			
**12.1**	Salsolanol; 4-(4′-hydroxy-2′-methylcyclopent-2′-enyloxy)- 4-methylcyclopent-2-enol	The aerial parts of *S. villosa* Delile. ex Schul.	^91^
**12.2**	Sulphurous acid, isohexyl 2- pentyl ester	By GC-MS analysis of the aerial parts of *S. tetrandra*	^71^

#### 4.1.6. Flavonoids and isoflavonoids

**Figure 6.**
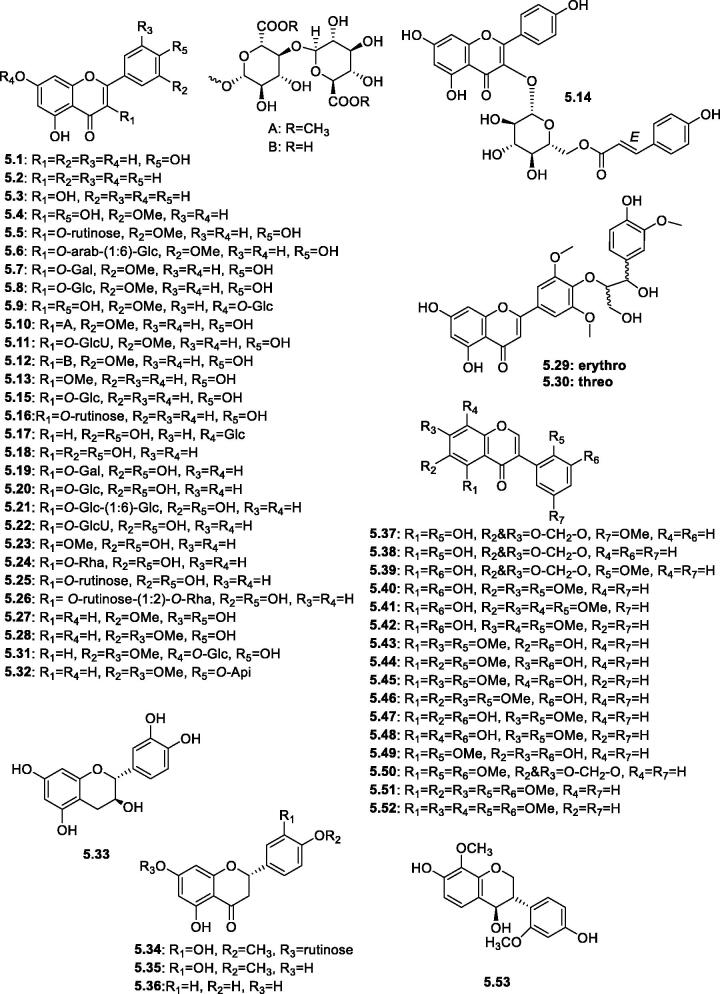
Structures of flavonoids and isoflavonoids derivatives (**5.1**–**5.53**) reported in the genus *Salsola.*

Flavonoids and isoflavonoids are predominant plant polyphenols having a C_6_-C_3_-C_6_ skeleton and are considered as one of the frequently studied plant phytochemicals^94^. Flavonoids are yellow-colored compounds possessing a highly distinctive biosynthetic pathway as they are synthesised from the mixed phenylpropanoid (4-coumaroyl-CoA) and polyketide (3 malonyl-CoA) pathway^95^. The isoflavonoids subclass is characterised by the presence of a 2-phenyl instead of 3-phenyl substitution at the benzo-γ-pyrone moiety^94^. Concerning the biological activities, flavonoids are the main dietary antioxidants due to their action as scavengers of harmful free radicals. In addition, they act as signalling molecules by their modulatory effect on several protein kinases, such as MAP kinase (mitogen-activated protein kinase). The latter mechanism can explain their neuroprotection, cardioprotection, and anticancer activities^96^. Isoflavonoids are much limited in their distribution in plant families (e.g. Leguminosae) compared to flavonoids and are characterised by their phytoestrogenic activity as in the case of genistein^97^. In the genus *Slasola*, the reported flavonoids (Figure 6) can be classified into flavones (such as apigenin **5.1**, chrysin **5.2**, luteolin-7-*O*-β-D-glucoside **5.17**, and tricin **5.28,** from *S. imbricata* Forssk, *S. kali* L., and *S. collina* Pall., respectively^60^^,^^66^^,^^75^^,^^84^, flavonols (such as isorhamnetin **5.4**, quercetin **5.18**, and kaempferol derivatives **5.13**–**5.16**), flavanols (such as catechin **5.33**), and flavanones (such as hesperidin **5.34**, hesperitin **5.35**, and naringenin **5.36**). The free flavonol aglycone, kaempferol was incompletely identified by UPLC/qTOF-MS analysis of the aerial parts and roots of *S. vermiculata* and *S. Tetrandra* plants^68^. The presence of OCH_3_ groups (i.e. methoxylated flavonoids) was mainly observed at C-3`and C-4′ in the B-ring of flavones (in tricin and its derivatives **5.28**–**5.32**), and at C-3′ of flavonols (in the isorhamnetin derivatives **5.4**–**5.13**). However, diversity in methoxylation positions was recorded for the isoflavonoids group (**5.37**–**5.52**), as both the A-ring (positions C-5, 6, 7, and 8) and the B-ring (positions C-2′, 3′, and 5′) acquired OCH_3_ groups. For detailed references and the plant source, see Table 2. Finally, a unique 8,2′-dimethoxylated isoflavan derivative, salisoflavan **5.53**, was reported from the arial parts *S. imbricata* Forssk^57^.

#### 4.1.7. Lignans

**Figure 7.**
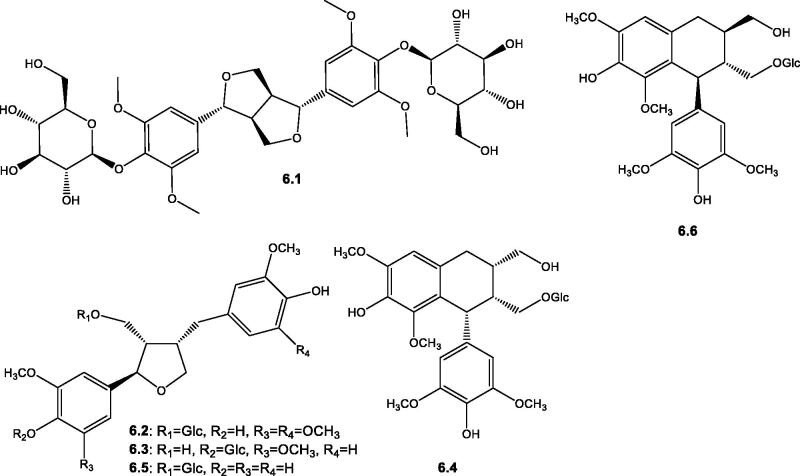
Structures of lignans (**6.1**–**6.6**) reported in the genus *Salsola.*

Lignans are natural secondary metabolites biosynthesized from the oxidative coupling of two *p*-hydroxyphenylpropane moieties (C_6_-C_3_) linked by a bond connecting the middle (β-β`) carbons of their side chains^98^. Regarding the genus *Salsola*, six derivatives from two major subclasses, lignans and cylolignans, were identified. For the lignans subclass, three tetrahydrofuran derivatives, alangilignoside C **6.2**, conicaoside **6.3**, and lariciresinol-9-*O*-β-D-glucopyranoside **6.5** were isolated from the aerial parts of *S. komarovii*^89^. Regarding the cylolignans subclass, two tetrahydronaphthalene derivatives, namely (8*S*,8`*R*,7`*R*)-9′-[(*β*-glucopyranosyl)oxy]lyoniresinol **6.4** and (+)-lyoniresinol 9′-*O*-*β*-D-glucopyranoside **6.6**, were isolated from the same plant^89^, Table 2 and Figure 7. In addition, another bicyclolignan derivative having a 3,7-dioxabicyclo[3.3.0]octane ring system, namely acanthoside D **6.1** was isolated from *S. collina* plant^60^.

#### 4.1.8. Triterpenoids and their derivatives

**Figure 8.**
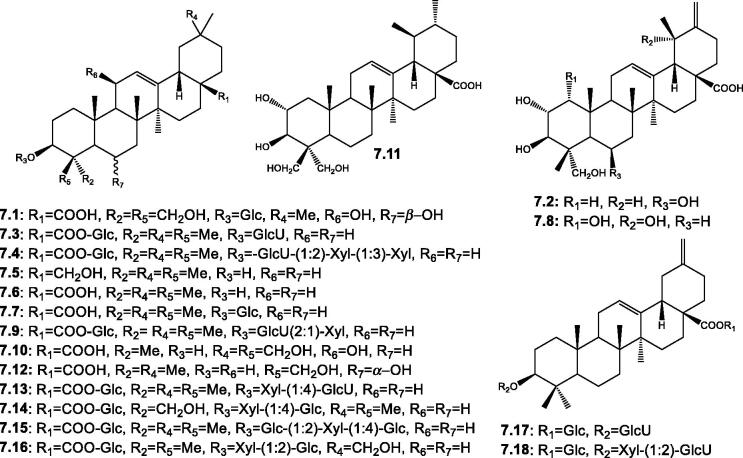
Structures of triterpenoids and nortriterpenoids (**7.1**–**7.18**) reported in the genus *Salsola.*

Triterpenoids are structurally diverse widely distributed natural phytochemicals possessing a C_30_-skeleton and are biosynthesized from the isoprenoid precursor, squalene^99^. Pentacyclic triterpenoids of the C–C–C–C(–C) 6–6-6–6-6 rings were reported in some *Salsola* spp. categorised as triterpenoids and nortriterpenoids (Table 2 and Figure 8). The triterpenoids group included mainly ursane, and oleanane skeletons, both free and combined. However, oleanane derivatives are the predominant group. Free hydroxylated oleanolic acid/derivatives are represented by guavenoic acid **7.2**, 1*α*,2*α*,3*β*,19*α*,23-pentahydroxyursa-12,20(30)-dien-28-oic acid **7.8**, salsolin A **7.10**, and salsolic acid **7.12** were isolated from *S. baryosma*^90^ and oleanolic acid **7.6** from *S. inermis* and *S. soda*^10^^,^^51^. Whereas, only olean-12-en-3,28-diol **7.5** found in *S. inermis* showed the presence of a primary alcoholic group (28-CH_2_OH) instead of a COOH at C-17 *^51^*. One ursane derivative, namely salsolin B **7.11** was identified from *S. baryosma*^90^. Concerning the reported combined triterpenoids, two positions of the triterpenoid's skeleton were noticed to possess sugar moieties; the first position is C-3 that showed the presence of a sugar chain of variable length ranging from 1–3 sugars (e.g. glucose, xylose, and glucuronic acid). The second one is C-28 which showed the presence of glucosyl esters. Of these saponins, three characteristic salsolosides were reported, including salsoloside C **7.13** from *S. micranthera* Botsch, *S. grandis* Freitag, Vural, and *S. soda*^10^^,^^62^^,^^70^^,^^77^, salsolosides D **7.14**, and E **7.15** from *S. micranthera* Botsch^63^. Two 3-β-hydroxy 30-noroleana-12,20(29)-dien-28-oic acid (syn. akebonic acid) derivatives were isolated from the roots of *S. imbricata* Forssk and identified as 3-*O*-*β*-D-glucuronopyranosyl-30-norolean-12,20(dien-28-*O*-[*β*-D-glucopyranosyl] ester **7.17** and 3-*O*-*β*-D-xylopyranosyl-(1 → 2)-*O*-*β*-D-glucuronopyranosyl-akebonic acid 28-*O*-β-D-glucopyranoside **7.18**^61^.

**Figure 9.**
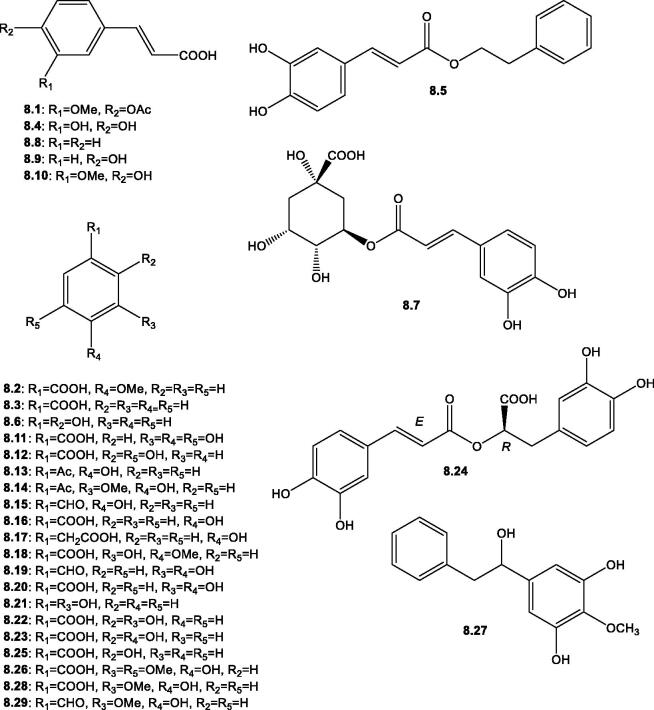
Structures of phenolic acids derivatives and simple phenols (**8.1**–**8.29**) reported in the genus *Salsola*.

#### 4.1.9. Phenolic acids and simple phenols

Simple phenols are a minor class of natural products defined as aromatic compounds with at least one hydroxyl group attached to a benzene ring, such as catechol, resorcinol, and phloroglucinol. However, phenolic acids/derivatives represent a major class of plant-derived natural products, categorised into benzoic acids, such as protocatechuic and gallic acids (C_6_-C_1_) and cinnamic acids, such as caffeic and coumaric acids (C_6_-C_3_)^100^. HPLC analysis of the aerial parts and root of *S. kali* revealed the presence of two simple phenols *viz*, catechol **8.6** and resorcinol **8.21**^12^^,^^75^. The presence of simple aromatic aldehydes was reported from *S. tuberculatiformis* Botsch. (4-hydroxybenzaldehyde **8.15**) and *S. collina* Pall. (protocatechuic aldehyde **8.19** and vanillin **8.29**)^66^. However, diverse benzoic acids were found in several plants of the genus *Salsola*, the most characteristic of which are gentisic acid **8.12**, *α*-resorcylic acid **8.22**, and *β*-resorcylic acid **8.23** from the herb and root of *S. kali*^12^, and the dihydrostilbene, tetranin A **8.27** from the roots of *S. tetrandra* Folsk^59^. In addition, various free cinnamic acids and their esters were reported from the plants of this genus. Regarding free cinnamic acids, previous phytochemical studies on *S.* kali, *S. imbricata* Forssk, *S. vermiculata*, *S. tetrandra*, *S. cyclophylla*, and *S. collina* Pall. showed the presence of caffeic **8.4**, cinnamic **8.8**, *p*-coumaric **8.9**, and ferulic acids **8.10**^12^^,^^15^^,^^66^^,^^68^^,^^80^^,^^84^. Whereas cinnamic acid esters were described in two *Salsola* spp. *viz*., *S. cyclophylla* and *S. imbricata* Forssk., including β-​phenylethyl caffeate **8.5**, chlorogenic acid **8.7**, and rosmarinic acid **8.24**^15^^,^^84^, Table 2 and Figure 9.

#### 4.1.10. Miscellaneous glycosides

**Figure 10.**
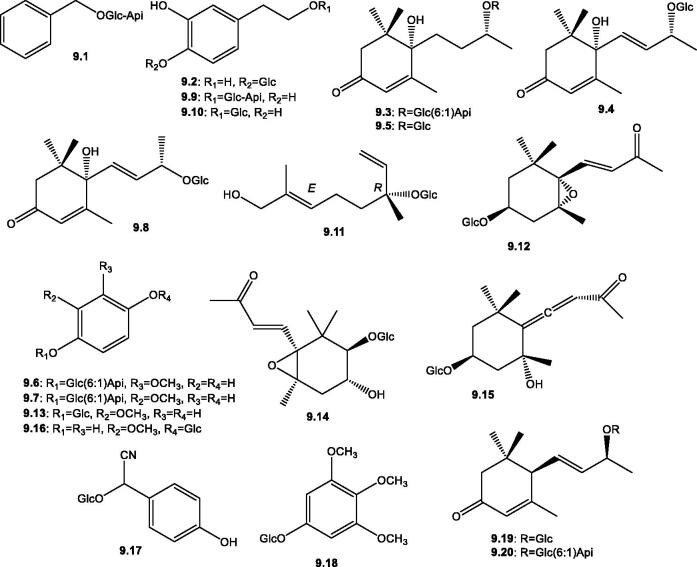
Structures of miscellaneous glycosides (**9.1**–**9.20**) reported in the genus *Salsola.*

Several miscellaneous glycosides with both phenolic and isoprenoid aglycones were reported from several plants of the genus *Salsola*. The glycone part in most cases is either glucose or *β*-D-apiofuranosyl-(1 → 6)-β-D-glucopyranose. The phenolic glycosides, benzyl 6-*O*-β-D-apiofuranosyl-β-D-glucopyranoside **9.1,** biophenol 2 **9.2,** cuneataside C **9.9**, and 2–(3,4-dihydroxy)-phenyl-ethyl-β-D-glucopyranoside **9.10** were isolated from the aerial parts of *S. komarovii*^89^. The cyanogenic glycosides, taxiphyllin **9.17** and 3,4,5-trimethoxyphenyl-*β*-D-glucopyranoside **9.18** were reported in the aerial parts of *S. tetrandra*^53^. Whereas the isoprenoid glycosides comprised the acyclic monoterpene, 9-​hydroxylinaloyl glucoside **9.11** from *S. tetrandra*^53^, in addition to several ionone derivatives with different unsaturation and oxidation status, such as roseoside A **9.4** and blumenyl B *β*-D-glucopyranoside **9.5** from *S. komarovii*^89^ and the epoxy derivatives icariside B2 **9.12** and lyohebecarpin A **9.14** from *S. komarovii* and *S. tetrandra*, respectively^53^^,^^89^ were reported, Table 2 and Figure 10.

**Figure 11.**
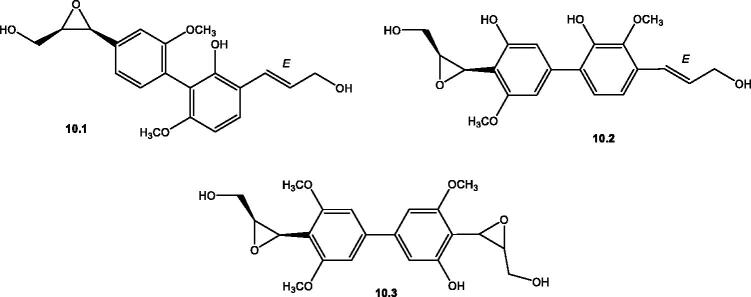
Structures of biphenylpropanoids (**10.1**–**10.3**) reported in the genus *Salsola.*

#### 4.1.11. Biphenylpropanoids

Biphenylpropanoids (Table 2 and Figure 11) were isolated from the aerial parts of *S. villosa* Delile. ex Schul. and the roots of *S. imbricata*. They are formed of dimeric C_6_C_3_ residues (linked head to head) with a characteristic oxirane ring formed by epoxidation of either one of the side chains' double bond as in biphenylsalsinol **10.1**^91^ and biphenylsalsonoid A **10.2**^92^ or both as in case of biphenylsalsonoid B **10.3**^92^.

#### 4.1.12. Polyhydric alcohols and carbohydrates

**Figure 12.**
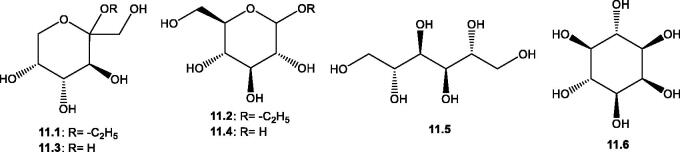
Structures of polyhydric alcohols and carbohydrates (**11.1**–**11.6**) reported from the genus *Salsola.*

Syrchina et al.^93^ described the presence of a few monosaccharide derivatives, including two simple ethyl glucosides namely, ethyl β-D-fructopyranoside **11.1** and ethyl β-D-glucopyranoside **11.2** from *S. collina* Pall. In addition, they reported the presence of two polyhydric alcohols (D-mannitol **11.5** and myoinositol **11.6**) from the same plant^93^, Table 2 and Figure 12.

**Figure 13.**
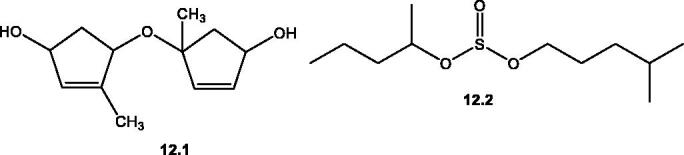
Miscellaneous compounds (**12.1**–**12.2**) reported in the genus *Salsola.*

#### 4.1.13. Miscellaneous group

Only two compounds are included in this group; the first one is a dimeric methylcyclopentenyl alcohol namely, salsolanol **12.1** isolated from the aerial parts of *S. villosa* Delile. ex Schul. ^91^. While, the second compound is an isohexyl 2- pentyl ester of sulphurous acid **12.2** detected by GC-MS analysis of the aerial parts of *S. tetrandra*^71^, Table 2 and Figure 13.

## 5. Pharmacological activities

Plants of the genus *Salsola* are widely used in the folk medicine of different countries for the treatment of several diseases, such as hypertension, broken bones as well as for boosting the immunity (Table 3).

**Table 3.** Traditional medical uses of *Salsola* species.

Country	S. sp.	Traditional use	Reference
China	*S. collina* Pall.	Treatment of hypertension, headache, and vertigo	^43^ ^,^ ^66^
Saharo-arabic and Soudano-deccanian	*S. baryosma*	Vascular hypertension	^101^
Middle East	*S. baryosoma*	Against inflammation and as a diuretic agent	^14^ ^,^ ^102^
Chhindwara, India	The whole plant of *S. kali* L.	Treatment of cough	^14^ ^,^ ^103^
Ethiopia	*S. somalensis*	Anthelmintic	^55^ ^,^ ^88^
Mongolia	Aerial parts of *S. laricifolia*	Used by the nomads of the Gobi Desert as winter tonic tea, for wound healing, and treatment of broken bones and swollen joints	^76^
Saudi Arabia	Leaves of *S. cyclophylla*	Used by local Bedouin as diuretic, laxative, anthelmintic, and anti-inflammatory	^15^ ^,^ ^41^
Turkmenistan, Tajikistan, and Kyrgyzstan	*S. richteri*	Used to treat skin conditions and hypertension in Tajik folk medicine	^76^
Southern Africa	Aqueous extract of *S. tuberculatiformis*	Used by Bushmen women as oral contraceptive	^40^ ^,^ ^104^

**Figure 14.**
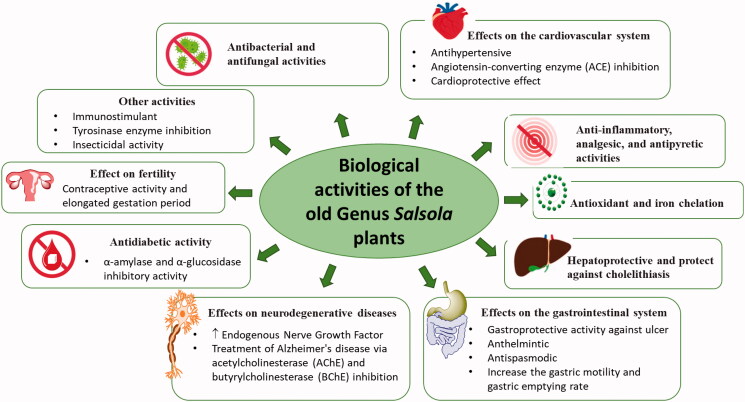
Reported pharmacological activities of plants belonging to the old genus *Salsola.*

Research studies showed that extracts of different *Salsola* spp. and compounds isolated from them exert a wide range of variable pharmacological activities. These activities will be discussed in detail in this section. They are also summarised in Table 4 and Figure 14.

**Table 4.** Reported pharmacological activities of *Salsola* species.

Pharmacological action/medicinal use	Salsola spp./part used	Extract /or product used	Collection place	Reference
**Effect on the cardiac system and blood pressure**				
Antihypertensive	*S. kali*, *S. longifolia,* and *S. ruthenic*	–	–	^81^ ^,^ ^105^
Angiotensin-converting enzyme inhibiting activity	Aerial parts of *S. oppositifolia,* and *S. soda*	Ethyl acetate extracts	Italy	^106^
Cardioprotective effect	Whole shrub of *S. kali*	Aqueous extract	New Damietta City, Egypt	^107^
**Anti-inflammatory, analgesic, and antipyretic activities**				
Anti-inflammatory and antinociceptive activities	Aerial parts of *S. grandis*	Ethanolic extract	Nallıhan bird sanctuary, Ankara, Turkey	^77^
Anti-inflammatory and analgesic activity	Aerial parts of *S. Cyclophylla*	Aqueous-ethanolic extract	Al-Fuwayliq City in the Qassim region, Saudi Arabia	^15^
Anti-inflammatory	*S. komarovii*	Ethanol extract	Yongin, Korea	^37^
Anti-inflammatory	Leaves of *S. imbricata* Forssk	Aqueous methanolic extract	Baharia Oasis, Egypt	^80^
Anti-inflammatory, analgesic, and antipyretic	Aerial parts of *S. imbricata*	Aqueous ethanol (30:70 v/v) extract	Cholistan desert, Punjab, Pakistan	^108^
**Antioxidant and Iron chelation activity**				
Antioxidant and Iron chelation activity	*S. cyclophylla*	Aqueous ethanolic Extract	Al-Fuwayliq City in the Qassim region, Saudi Arabia	^15^
Antioxidant	*S. Cyclophylla*	Essential oil	Qassim region, Saudi Arabia	^41^
Antioxidant	Leaves and stems of *S. kali* L.	Methanol extract	Borj-Cédria coastal Region, Tunis	^75^
Antioxidant activity	Aerial parts of *S. oppositofolia*, *S. soda*, and *S. tragus*	Alkaloid extract	Central and Southern Italy	^48^
Antioxidant activity	Aerial parts of *S. komarovii*	Ethyl acetate extract	Gangneung, Korea	^79^
Antioxidant	*S. baryosma*	Ethyl acetate fraction	Cholistan desert, Pakistan	^109^
Antioxidant	*S. baryosma*	80% (v/v) Aqueous methanol extract	Algeria	^22^
**Cytotoxic activity**				
Cytotoxic activity	Aerial parts of *S. oppositifolia* Desf.	Different extracts were tested	Sicily, Italy	^85^
Cytotoxic activity	*S. collina*	Ethanol extract	–	^110^
Phytotoxic activity	*S. baryosma*	Ethyl acetate fraction	Cholistan desert, Pakistan	^109^
**Effect on the liver and the gallbladder**				
Hepatoprotective effect	Aerial parts of *S. collina* Pall	25% Ethanol extract	Russia	^111^
Anti-cholelithiasis	*S. collina* Pall	Aqueous extract	Russia	^112^
Hepatoprotective effect	Aerial parts of *S. tetrandra*	70% Hydroalcoholic extract	Saudi Arabia	^113^
Hepatoprotective and antioxidant effect	Whole plants of *S. imbricata* Forssk	Ethanolic and methanolic extracts	Muhaisnah desert, Dubai, UAE	^84^
Hepatoprotective effect	Aerial parts of *S. tetrandra* and *S. baryosma*	70% Ethanol-water	Saudi Arabia	^113^
Hepatoprotective effect	*S. villosa* and *S. volkensii*	Aqueous-alcoholic extract	Egypt	^114^
**Effects on the gastrointestinal system**				
Gastroprotective	*S. komarovii*	50% Alcohol extract	Korea	^115^
Gastroprotective	*S. tetrandra*	70% Alcoholic extract	El Doubia at ElRiyadh- El Dallamroad, Saudi Arabia	^71^
Anthelmintic Activity	Bark of *S. imbricata*	Chloroform extract	Bahawalpur District, Pakistan	^116^
Antispasmodic	*S. baryosma*	Ethyl acetate fraction	Cholistan desert, Pakistan	^109^
Antispasmodic and bronchorelaxant activities	Aerial parts of *S. imbricata*	Aqueous-ethanol extract	Cholistan desert, district Bahawalpur, Pakistan	^117^
Improving gastric emptying	*S. collina*	Ethyl acetate extract	–	^118^
**Antidiabetic activity**				
α-amylase inhibitory activity	*S. kali*	Ethyl acetate fraction	Calabria, Italy	^65^
Moderate α-amylase inhibitory activity	Whole plant of *S. collina* Pall	*N*-Acetyltryptophan isolated from 80% EtOH extract	Shandong province, China	^66^
α-Glucosidase and α-Amylase enzyme inhibitory	*S. vermiculata* and *S. baryosma*	Phenolic extract	Algeria	^22^
Aldose reductase inhibition	Aerial parts and cultivated buds of wild *S. soda*	The *n*-BuOH extracts	Pisa, Italy	^10^
**Effect on neurodegenerative diseases**				
Nerve growth factor induction	Aerial parts of *S. komarovii*	80% Methanol extract	Jejudo, Korea	^89^
Anti-Alzheimer's, and antioxidant activity	Aerial parts of *S. oppositofolia*, *S. soda*, and *S. tragus*	Alkaloid extract	Central and Southern Italy	^48^
Acetylcholinesterase inhibitory activity	Root of *S. vermiculata*	Methanol extract	Marsa Matrouh, Egypt	^68^
Acetylcholinesterase inhibitory activity	Aerial parts of *S. grandis*	96% EtOH extract	Ankara, Turkey	^70^
Butyrylcholinesterase inhibitory activity	*S. baryosma*	Chloroform extract	Pakistan	^90^
**Contraceptive activity**				
Contraceptive effect on Female sheep and rats	*S. tuberculatiformis*	96 % Ethanol extract	South West Africa	^119^
Contraceptive effect on male rats	*S. imbricata* Forssk	Ethanol extract	Muhaisnah Desert, Dubai, UAE	^104^
**Effect on melanin biosynthesis**				
Tyrosinase enzyme inhibitory activity	*S. foetida*	*Trans*-*N*-feruloyltyramine derivatives	Lal Sohanra National Forest Park of Bahawalpur, Pakistan	^78^
**Antimicrobial activity**				
Antibacterial	Aerial parts of *S.* *villosa*	Chloroform extract and isolated compounds	Arar, Saudi Arabia	^91^
Antibacterial	Roots of *S. imbricata*	Biphenylsalsonoids A and B	Arar, Saudi Arabia	^92^
Antibacterial	*S. kali* L. stem	Methanol extract	Borj-Cédria coastal Region, Tunis	^75^
Antibacterial and antifungal activities	*S. cyclophylla*	Essential oil	Qassim region, Saudi Arabia	^41^
Antibacterial	Roots of *S. vermiculate*	Ethanolic extract	Monastir, Tunisia	^42^
Antifungal activity	Aerial parts of *S. vermiculate*	Aqueous extract	kanadssa Bechar, Algeria	^120^
Antifungal activity	*S. collina* Pall	Terrestric acid	Shandong province, China	^66^
**Insecticidal activity**				
against *Trogoderma granarium*	Leaves of *S. baryosma* (schultes)	Ethanol extract	Pakistan	^121^

### 5.1. Effect on the cardiac system and blood pressure

One of the early reported pharmacological activities of *Salsola* spp. is their antihypertensive action. Different *Salsola* spp. are used as ingredients in different Chinese patents obtained from Faming Zhuanli Shenqing for treating hypertension. Of these, *S. collina* was the most extensively used sp. as indicated by the number of patents addressed this particular plant. Also, *S. ruthenica* and *S. arbuscula* were used in some Chinese patents. Likewise, *S. ruthenic*, a synonym for *S. tragus*, was reported as a potential treatment for essential hypertension^105^. Ammon et al.^81^ attributed the antihypertensive activity of *S. kali* and *S. longifolia* Forsk to salsoline **1.16** and salsolidine **1.19** alkaloids due to their ability to stimulate respiration and to decrease blood pressure^81^.

Loizzo et al.^106^ investigated the inhibitory activity of different extracts of the aerial parts of *S. oppositifolia* Desf., *S. soda* L., and *S. tragus* against the angiotensin-converting enzyme (ACE). The ethyl acetate extracts of *S. oppositifolia* and *S. soda* showed interesting activities with IC_50_ values of 181.04 and 284.27 µg/mL, respectively which further support the traditional antihypertensive use of these species.

The aqueous extract of the whole shrub of *S. kali* was reported to display a cardioprotective effect against adriamycin-induced cardiotoxicity in male Swiss albino mice^107^. This effect was attributed to lowering the oxidative stress in the heart and inhibiting lipid peroxidation^107^.

### 5.2. Anti-inflammatory, analgesic, and antipyretic activities

Janbaz et al.^108^ tested the aqueous-ethanol extract of the aerial parts of *S. imbricata* to assess its traditional use in inflammatory conditions. They confirmed the anti-inflammatory activity of *S. imbricata* as it significantly inhibited carrageenan-induced paw edoema in rats. The same research group also tested the analgesic activity of *S. imbricata* extract using NaCl-induced writhing and formalin-induced paw licking models in rats. Their obtained results indicated that *S. imbricata* exhibited a dose-dependant analgesic activity by reducing the number of abdominal writhing mediated by 4% NaCl intraperitoneal injection at all tested doses (100, 300, and 500 mg/kg)^108^. Nevertheless, it decreased the time of paw licking by rats only at the dose of 500 mg/kg. Also, *S. imbricata* showed significant antipyretic activity in the brewer’s yeast-induced pyrexia model in rats^108^. The aqueous methanolic extract of *S. imbricata* leaves and the phenolic compounds isolated from it decreased the NO production levels in *in-vitro* LPS-induced inflammation in RAW 264.7 macrophage cells and were found to be non-toxic at the concentration of 100 µg/mL^80^. Regarding the tested phenolic compounds, isorhamnetin-3-*O*-glucopyranoside **5.8** displayed higher activity than its corresponding galactopyranoside glycoside **5.7** and aglycone **5.4**^80^.

The anti-inflammatory and antinociceptive activities of *S. grandis* were tested using the carrageenan-induced paw edoema model in rats and *p*-benzoquinone-induced nociception tests in mice, respectively^77^. The ethanolic extract obtained from the aerial parts of *S. grandis* was fractionated and the most bioactive fraction (*n*-BuOH) was further subjected to a bioassay-guided fractionation to isolate the compounds responsible for *S. grandis’s* activity. The flavonoidal compounds, tiliroside **5.14** and quercetin-3-*O*-β-D-galactoside **5.19** displayed the highest activities in the used models^77^.

The anti-inflammatory activity of different extracts of the aerial parts of *S. cyclophylla* was evaluated by Mohammed et al.^15^ using the carrageenan-induced paw edoema method. The aqueous-ethanolic extract showed the highest anti-inflammatory activity among the tested extracts and its activity was close to the well-known anti-inflammatory drug, diclofenac. Mohammed et al. attributed this anti-inflammatory activity to the antioxidant potential of the phenolic and flavonoid components present in the aqueous-ethanolic extract. The same research group also investigated the analgesic activity of *S. cyclophylla* using the hot-plate and acetic-acid writhing models in mice. The aqueous ethanolic extract showed the highest activity with 87.50– 99.66% pain reduction rates after different time intervals, which was comparable to the diclofenac activity^15^.

Seo et al.^37^ reported that the ethanol extract of *S. komarovii* showed effective anti-inflammatory activity as hydrocortisone by reducing the production of LPS-induced IL-6. It also exerted glucocorticoid receptor binding activity and interfered with NF-κB nuclear translocation^37^.

The synthetic analogue of the active principle of *S. tuberculata*, 2–(4-acetoxyphenyl)2-chloro-*N*-methylethylammonium-chloride, was reported to inhibit UVB induced intracellular interleukin-1 alpha (icIL-1α) in the UVB *in-vitro* model for inflammation^122^. Contrarily, the methanol extract of *S. tuberculata* exerted a pro-inflammatory activity by boosting the UVB induced-icIL-1α production and enhanced cytotoxicity. While the dichloromethane extract showed no significant effect on skin cells inflammation^122^. The investigated synthetic analouge was also suggested to exert its anti-inflammatory and contraceptive activities by competitive inhibition of glucocorticoid binding to corticosteroid-binding globulin (CBG) leading to increased levels of the *in-vivo* free corticosterone^123^^,^^124^.

### 5.3. Antioxidant and iron chelation activities

The antioxidant potential is one of the most extensively studied activities of *Salsola* species. It could be concluded from the reported results that the used plant parts and the extraction solvent could greatly affect the antioxidant activity. Flavonoids and their glucosidal derivatives are mostly the responsible compounds for antioxidant activities. While other compounds, such as essential oil components, alkaloids, and biphenylpropanoids showed only moderate activities.

The antioxidant activity of *S. cyclophylla* extracts was tested using 2,2-diphenyl-1-picrylhydrazyl (DPPH) colorimetric assay method^15^. The best DPPH-free radicals scavenging potential was observed for the aqueous-ethanolic extract that showed comparable activity to the used standard, quercetin. While, the ethyl acetate extract showed the highest ferrous ions (Fe^2+^) chelating activity using ferrozine-based assay^15^. The same group reported the antioxidant activity of the essential oil obtained by water distillation of *S. cyclophylla* that showed only one-half of the quercetin activity. They attributed this activity to the benzoic acid esters and the hexahydrofarnesyl acetone components that occur in the essential oil in high concentrations^41^.

Antioxidant and iron chelation activities of the methanolic extract of different plant parts of *S. kali* were also investigated by Boulaaba et al.^75^ using the same methods used for *S. cyclophylla* extracts. Leaf and stem extracts showed the highest antioxidant activity while leaf and root extracts showed the highest iron chelation activity^75^.

The alkaloidal extracts of *S. oppositofolia*, *S. soda*, and *S. tragus* were prepared by extraction of their aerial parts with methanol, alkalinization with NH_4_OH then extraction with ethyl acetate. The three alkaloidal extracts showed significant antioxidant activity when tested using the DPPH method. Remarkably, *S. oppositifolia* showed the highest activity with an IC_50_ value of 16.30 µg/mL^48^.

Oueslati et al.^92^ investigated the antioxidant activity of biphenylsalsonoids A (**10.2**) and B (**10.3**) isolated from the ethyl acetate fraction of the roots of *S. imbricata* using DPPH and 2,2′-azinobis(3-ethylbenzothiazoline-6-sulfonic acid (ABTS^+^) assay methods. The two compounds showed moderate antioxidant activity^92^. *Trans*-*N*-feruloyltyramine derivatives isolated from *S. foetida* (**1.3**, **1.4**, and **1.8**) exhibited moderate antioxidant activity with IC_50_ ranging from 378 to 427 µM using DPPH radical scavenging assay^78^.

The ethyl acetate extract of *S. komarovii* aerial parts was subjected to HPLC separation and the obtained elutes were tested for antioxidant activity using ABTS^+^ radical scavenging method. The components responsible for the antioxidant activity were identified by HPLC-MS as the flavonoids, isorhamnetin **5.4**, astragalin **5.15**, isoquercitrin **5.20**, and rutin **5.25**^79^.

The ethyl acetate fraction of *S. baryosma* showed 77% DPPH radicals scavenging activity while other tested fractions showed lower activities below 57%^109^. This result is contradictory with that obtained by Khacheba et al.^125^ who reported weak antioxidant activity of *S. baryosma* ethyl acetate extract using DPPH assay.

The antioxidant activity of 80% (v/v) aqueous-methanol extracts of *S. vermiculata* and *S. baryosma* in addition to other Algerian herbs was tested using the hydroxyl (OH^•^), nitroxide (NO^•^) and (ABTS^+^) radicals scavenging assays, and Fe^3+^–TPTZ complex reductive power assay. The results showed that *S. baryosma* exhibited the highest antioxidant activity in OH^•^ radical assay with an EC_50_ of 0.26 ppm despite its low phenolic content^22^.

Beyaoui et al.^59^ investigated the antioxidant activity of two compounds, tetranins A and B, isolated from the ethyl acetate extract of *S. tetrandra* roots using DPPH and ABTS assays. The dihydrostilbene, tetranin A **8.27** exerted higher antioxidant activity than the isoflavonoid, tetranin B **5.37.** However, both compounds showed lower activity than the standard antioxidant, Trolox^59^.

The ethanol extract of *S. collina* Pall demonstrated anti-oxidative activity through DPPH radical scavenging capacity (Oh et al., 2014).

### 5.4. Cytotoxic activity

Only a few studies were made for investigating the cytotoxic activity of a small number of *Salsola* spp., including *S. cyclophylla*, *S. oppositifolia*, *S. collina* Pall, and *S. baryosma*. The cytotoxic activity of 95% aqueous-ethanolic extract of the aerial parts of *S. cyclophylla* was investigated using MTT assay against M14 melanoma derived epithelial breast cancer (MDA cells), human pancreatic cancer (PANC-1), Michigan Cancer Foundation-7 (MCF-7) breast cancer cells, and the normal human fibroblast cells. The aqueous-ethanolic extract of *S. cyclophylla* showed low to moderate cytotoxic activity only at high concentrations (50–400 µg/mL) against the tested cell lines and no significant cytotoxic effect was observed at low concentration (< 50 µg/mL)^15^.

Different fractions obtained from the extract of the aerial parts of *S. oppositifolia* were screened for cytotoxic activity against a panel of cancer cell lines^85^. The *n*-hexane fraction showed the highest cytotoxic activity on lung carcinoma (COR-L23) and amelanotic melanoma (C32) cell lines with IC_50_ values of 19.1 μg/mL and 24.4 μg/mL, respectively. The dichloromethane fraction also demonstrated cytotoxic activity against these two cell lines with IC_50_ values of 30.4 μg/mL and 33.2 μg/mL for COR-L23 and C32 cell lines, respectively. The ethyl acetate fraction exhibited a selective moderate cytotoxic activity against breast cancer, MCF-7 cells (IC_50_ 67.9 μg/mL). The major constituents isolated from the ethyl acetate fraction, isorhamnetin-3-*O*-glucopyranoside **5.8** and isorhamnetin-3-*O*-rutinoside **5.5** also demonstrated a potential activity against MCF-7 with IC_50_ values of 18.2 and 25.2 μg/mL, respectively. Additionally, isorhamnetin-3-*O*-rutinoside **5.5** showed high activity against the hormone-dependent prostate carcinoma cell line (LNCaP) with an IC_50_ value of 20.5 μg/mL^85^.

The ethanol extract of *S. collina* Pall showed cytotoxic activity against human colon carcinoma cells (HT29). It resulted in a reduction in the number and size of the cells through cell cycle regulation and caused cell arrest in the G2/M phase^110^.

The ethanol extract of *S. baryosma* whole plant showed no significant cytotoxic activity when tested with other plant extracts using the brine shrimp method^126^. The same result was reported by Ahmed et al.^109^, while 80% ethanol extract of *S. baryosma* did not exhibit cytotoxic activity against brine shrimp larvae and only the ethyl acetate fraction showed 50% cytotoxic activity. However, all tested fractions of *S. baryosma* showed phytotoxicity against *Lemna minor* plant growth^109^.

### 5.5. Effect on the immune system

Interestingly, *S. laricifolia* Turcz is reported to be one of the immune system-boosting drugs, and a pharmaceutical product derived from it “Salimon” represents one of the best-selling immunostimulant drugs in the Mongolian drug market^76^.

### 5.6. Effect on the liver and the gallbladder

Lochein, a liquid extract of the Russian thistle *S. collina* Pall., was reported to show a significant hepatoprotective effect on patients with chronic hepatitis^127^. It also has been approved as an active food supplement by the Ministry of Health of the Russian Federation^111^. Ethanol extract (25%) of the aerial parts of *S. collina* Pall. was reported to decrease the signs of paracetamol-induced liver damage in rats and to exert a better hepatoprotective activity than the reference drug, silymarin^111^. It was also reported to decrease the levels of the liver enzymes and lipid peroxidation products and to enhance the detoxification of bilirubin, and ammonia^111^. Moreover, *S. collina* aqueous extract was reported to protect against cholelithiasis in rabbits through enhancing cholesterol and water absorption and decreasing inflammation and formation of biliary slough^112^.

Oral administration of *S. imbricata* methanol extract was reported to prevent liver toxicity in CCl_4_-induced hepatotoxicity in mice. This hepatoprotective activity was attributed to the ability of the phenolic content of *S. imbricata* to enhance the antioxidant capacity of the liver^84^.

Ethanol extracts (70%) of *S. tetrandra* and *S. baryosma* showed a prophylactic and therapeutic hepatoprotective activity against paracetamol-induced hepatorenal toxicity in rats^113^. The results showed that *S. tetrandra* was more active and showed a higher ability to decrease the levels of inflammatory markers, such as interleukin-1β (IL-1β) and tumour necrosis factor alpha (TNF-α)^113^.

The alcoholic extracts of *S. volkensii* and *S. villosa* showed hepatoprotective effects with a broad safety margin against CCl_4_-induced hepatotoxicity in Sprague Dewaly rats indicating their potential use for the treatment of liver damage^114^,.

### 5.7. Effects on the gastrointestinal system

Different plants of the *Salsola* genus were reported to exert several effects on the gastrointestinal tract, including gastroprotective activity against ulcer, anthelmintic, and antispasmodic activities.

Alcoholic extract (50%) of *S. komarovi* in 500 mg/kg concentration was found to significantly protect against gastric ulcer and to be more potent than Ranitidine (300 mg/kg) in 60% HCl-ethanol induced gastritis model^115^. While 70% alcoholic extract of *S. tetrandra* showed a similar gastroprotective effect to that of Ranitidine against aspirin-induced gastric ulceration in rats^71^.

Chloroform extract of *S. imbricata* bark demonstrated anthelmintic activity against *Haemonchus contortus* worms^116^. Ethanol extract (80%) of *S. baryosma* (synonym for *S. imbricata*) demonstrated antispasmodic activity as it inhibited the rabbit jejunum contraction at a concentration of 0.3–3 mg/mL^109^. It was suggested to act as a calcium channel blocker because it resulted in 70% inhibition of K^+^-induced contractions in rabbit jejunum at the concentration of 1–5 mg/mL^109^. The ethyl acetate fraction of the aerial parts extract of the same sp. showed the highest spasmolytic and bronchorelaxant activities on isolated rabbit jejunum and tracheal preparations which were suggested to be due to its agonist action on β-adrenergic receptors and Ca^+2^ antagonising activity^117^.

On the other hand, the ethyl acetate extract of *S. collina* was reported to increase the gastric motility and gastric emptying rate through activating M-cholinergic receptor, increasing ghrelin and gastrin plasma levels and increasing the expression of the vasoactive intestinal peptide receptors in rats^118^^,^^128^.

### 5.8. Antidiabetic activity

Decreasing post-prandial hyperglycaemia by inhibiting digestive enzymes involved in carbohydrate hydrolysis, such as α-amylase and α-glucosidase enzymes is a commonly used therapeutic approach for the management of diabetes. Therefore extensive studies were made on the α-amylase and α-glucosidase inhibitory activity of different *Salsola* spp.^22^^,^^65^.

The α-amylase inhibitory activity of different fractions of the aerial parts of *S. kali*, *S. soda*, and *S. oppositifolia* was investigated by Tundis et al.^65^. The ethyl acetate fraction of *S. kali* showed the highest α-amylase inhibitory activity with an IC_50_ value of 0.022 mg/mL. The bioassay-guided chromatographic separation of this most active fraction resulted in the isolation of two flavonol glycosides, of which isorhamnetin-3-*O*-rutinoside **5.5** displayed significant α-amylase inhibitory activity with an IC_50_ value of 0.129 mM^65^.

Djeridane et al.^22^ investigated the antidiabetic potential of the aqueous-methanol extracts of *S. vermiculata* and *S. baryosma* by testing their ability to inhibit α-amylase and α-glucosidase enzymes activities. The results indicated that *S. baryosma* exhibited the highest competitive inhibitory activity with inhibition constant (K_i_) values of 7 and 16 µM against α-amylase and α-glucosidase, respectively suggesting its potential for type 2 diabetes management^22^. Similarly, *N*-acetyltryptophan **1.1** isolated from *S. collina* Pall by Jin et al.^66^ showed 44% inhibition of α-amylase enzyme activity.

Iannuzzi et al.^10^ studied the chemical profile of the cultivated buds of *S. soda* and compared it to that of the wild plant. They also screened the inhibitory activity of the compounds isolated from their *n*-BuOH fraction against three enzymes of the aldo/keto reductase superfamily, namely aldose reductase (hAKR1B1), aldose-reductase-like protein (hAKR1B10), and carbonyl reductase 1 (hCBR1). They found that quercetin-3-​*O*-​glucuronopyranoside **5.22**, the only flavonoid identified in both plant types was the most effective inhibitor for the tested enzymes and suggested its use as a functional nutraceutical to counteract diabetic complications^10^.

### 5.9. Effect on neurodegenerative diseases

The effect of the isolated compounds from the methanol extract of *S. komarovii* aerial parts on the production of the endogenous Nerve Growth Factor (NGF) in C6 glioma cells was investigated by Cho et al.^89^. The lignan derivative, conicaoside **6.3** showed the highest NGF-production stimulating activity and the lowest toxicity among the tested compounds indicating its potential for the regulation of neurodegenerative diseases, such as Alzheimer’s and Parkinson’s diseases^89^. Alzheimer’s disease (AD) is one of the most common neurodegenerative diseases that is combined with acetylcholine deficiency. Therefore, it can be improved by inhibiting the enzymes affecting the cleavage of acetylcholine, such as acetylcholinesterase (AChE) and butyrylcholinesterase (BChE).

The ethanolic extract of the aerial parts of *S. grandis* and the different compounds isolated from its *n*-BuOH sub-extract were investigated for AChE inhibitory activity by Orhan et al.^70^. Only *N*-acetyltryptophan **1.1** showed AChE inhibitory activity suggesting its neuroprotective potential against Alzheimer’s disease^70^.

The methanolic extract of *S. vermiculata* root demonstrated strong anti-acetylcholinesterase inhibitory activity which was higher than that of *S. vermiculata* aerial parts and *S. tetrandra* roots and aerial parts. It showed an IC_50_ of 0.45 ± 0.17 mg/mL. While the standard drug, eserine showed IC_50_ of 0.27 ± 0.1 mg/mL^68^. This activity could be attributed to the rich catecholamines content in *S. vermiculata* root^68^.

The alkaloidal extracts of *S. tragus, S. soda,* and *S. oppositifolia* Desf. were screened for AChE and BChE inhibitory activities^48^. *S. tragus* showed the highest inhibitory activity with IC_50_ of 30.2 and 26.5 µg/mL against AChE and BChE, respectively. While *S. soda* and *S. oppositifolia* Desf. showed selective inhibition of BChE with IC_50_ values of 34.3 and 32.7 µg/mL, respectively^48^. Salsolic acid **7.12** and other two triterpenes **7.2** & **7.8** isolated by Ahmad et al.^90^ from the chloroform extract of *S. baryosma* were reported to inhibit the BChE enzyme^90^.

### 5.10. Effect on fertility

The contraceptive activity of *Salsola* plants was firstly described by Ploss in 1960. He reported the use of the aqueous extract of an undefined *Salsola* sp. as an oral contraceptive in Algeria^40^. The aqueous extract of *S. tuberculatiformis* (previously known as *S. tuberculate* and commonly known as Gannabos) was reported to be used by Bushmen women as an oral contraceptive and to cause prolonged gestation and foetal post-maturity in Karakul sheep in Namibia region, South Africa^40^^,^^119^^,^^129^. Swart et al.^40^ investigated the phytochemicals responsible for this activity in *S. tuberculatiformis.* The compound responsible for this activity was reported to be a labile synephrine analogue with a reactive aziridine group. Therefore, they synthesised the compound, 2–(4-acetoxyphenyl)2-chloro-*N*-methylethylammonium-chloride, as a stable analogue for the active principle of *S. tuberculatiformis.* This compound was found to disturb the mammalian steroid hormones homeostasis and to inhibit adrenal steroidogenesis^40^.

The ethanolic extract of *S. imbricata* was reported to cause a slight decrease in the testis weight and to cause a significant decline in the sperm count when administered orally to male albino rats suggesting its potential use as a reversible male contraceptive, with a high safety margin^104^. They attributed this contraceptive activity to the phenolic content of the plant, especially quercitrin^104^.

### 5.11. Effect on melanin biosynthesis

*Trans*-*N*-feruloyltyramine derivatives (**1.3**, **1.4**, and **1.8**) isolated from *S. foetida* were reported to exhibit significant tyrosinase enzyme inhibitory activity with IC_50_ ranging from 0.40–2.61 µM which was lower than that of the standard tyrosinase inhibitors, kojic acid and L-mimosine, with IC_50_ of 16.67 and 3.68 µM, respectively. Therefore, these derivatives could have promising activities on melanocytes and skin pigmentation abnormalities^78^.

### 5.12. Antimicrobial activity

The chloroform extract of the aerial parts of *S. villosa* and the compounds isolated from it were tested against different bacterial strains using the paper disc diffusion method^91^. The isolated compound biphenylsalsinol **10.1** showed the highest antimicrobial activity against *Staphylococcus epidermidis*, *Staphylococcus aureus*, *Escherichia coli*, and *Pseudomonas aeruginosa* bacterial strains with an inhibitory zone diameter (IZD) ranging from 12.33 to 28.66 mm. While salsolanol **12.1** showed slight activity against *S. aureus*, *E. coli, S. epidermidis* with IZD ranging from 9.33 to 12.66 mm^91^. Oueslati et al.^92^ also investigated the antibacterial activity of the roots of *S. imbricata* and the bioactive compounds, biphenylsalsonoid A **10.2** and B **10.3**, isolated from its ethyl acetate fraction. The two isolated compounds showed similar antibacterial activity against *S. aureus*, *S. epidermidis* and *E. coli* with MIC values ranging from 16–32 µg/mL^92^. While biphenylsalsonoid A **10.2** was two times more active than biphenylsalsonoid B **10.3** against *Micrococcus luteus*. It is worth noting that both compounds showed lower activity than the standard drug, Kanamycin which showed MIC values ranging from 2–8 µg/mL^92^

The antimicrobial activities of the methanol extract of *S. kali* leaves and stems were investigated by Boulaaba et al.^75^. The stem extract showed higher activity than the leaf extract. It showed antibacterial activity against *P. aeruginosa* and *M. luteus* with an inhibition zone diameter (IZD) of 10 mm. It showed weak or slight activity against other bacterial pathogens and *Candida* sp.^75^.

Mohammed et al.^41^ investigated the antimicrobial activity of *S. cyclophylla* essential oil against different microorganisms using the agar well-diffusion method. It showed good antibacterial activity against the Gram + ve, *S. aureus* and *Streptococcus pyogenes*, and the Gram -ve, *P. aeruginosa*, and *E. coli*. However, it had no activity against *S. epidermidis*. It also demonstrated powerful antifungal activity against *C. albicans*^41^.

Gannoun et al.^42^ investigated the antimicrobial activities of *S. vermiculate* leaf, root, and stem extracts and their volatile fractions towards different pathogens. They reported that the ethanolic roots extract showed the highest activity against *S. aureus* with a MIC value of 0.28 mg/mL^42^. The used extracts showed low antifungal activity against the tested fungal sp. with IZD ranging from 6–9.5 mm^42^. On the other hand, *S. vermiculata* aqueous extract was reported to be an effective antifungal agent that can be used as a preservative during grain storage. This activity was examined by the decrease of fungal growth on wheat samples that were coated with *S. vermiculata* aqueous extract, dried, and stored for one year^120^.

Terrestric acid **1.20** isolated from *S. collina* Pall by Jin et al.^66^ showed antifungal activity against *Candida albicans* with a minimum 80% inhibitory concentration (MIC_80_) of 8 µg/mL^66^. The alkaloid salsoline A (trolline) **1.17**, present in *S. collina* Pall. and the flowers of *Trollius chinensis*, was reported to exhibit significant antibacterial activity against *S. aureus*, *Streptococcus pneumoniae*, and *Klebsiella pneumoniae*. It also exhibited moderate antiviral activity against influenza viruses A and B^46^.

### 5.13. Insecticidal activity

The ethanol extract of *S. baryosma* was reported to cause moderate insecticidal activity (22.08% mortality) against *Trogoderma granarium* insects (Everts) which was lower than the standard insecticidal compound, cypermethrin (37.64% mortality)^121^.

## 6. Conclusion

The impressive diversity of the pool of phytochemicals of *Salsola* spp. is comprehensively studied in this review. Furthermore, up-to-date taxonomic classification and description of the important morphological characteristics of the plants of this genus were discussed herein. The phytochemical profile of *Salsola* spp. is composed of alkaloids, nitrogenous compounds, flavonoids and isoflavonoids, triterpenoids, cardenolides and steroids, coumarins, coumarolignans, lignans and diphenylpropanoids, and simple phenolic acids. These secondary metabolites represent a great interest for the chemotaxonomy of the genus. Furthermore, they would support the diverse traditional medicinal uses and pharmacological activities of *Salsola* species demonstrated by many reports as antihypertensive, immunostimulant, anti-inflammatory, hepatoprotective, anthelmintic, antispasmodic, and antidiabetic. The current study represents a guiding light for researchers studying such widely distributed wild medicinal plants.
